# Oxaliplatin-induced changes in microbiota, TLR4+ cells and enhanced HMGB1 expression in the murine colon

**DOI:** 10.1371/journal.pone.0198359

**Published:** 2018-06-12

**Authors:** Vanesa Stojanovska, Rachel M. McQuade, Sarah Fraser, Monica Prakash, Shakuntla Gondalia, Rhian Stavely, Enzo Palombo, Vasso Apostolopoulos, Samy Sakkal, Kulmira Nurgali

**Affiliations:** 1 College of Health and Biomedicine, Institute for Health and Sport, Victoria University, Melbourne, Victoria, Australia; 2 Centre for Human Psychopharmacology, Swinburne University of Technology, Hawthorn, Melbourne, Victoria, Australia; 3 Department of Chemistry and Biotechnology, Swinburne University of Technology, Hawthorn, Melbourne, Victoria, Australia; 4 Department of Medicine Western Health, The University of Melbourne, Regenerative Medicine and Stem Cells Program, Australian Institute for Musculoskeletal Science (AIMSS), Melbourne, Victoria, Australia; "INSERM", FRANCE

## Abstract

Oxaliplatin is a platinum-based chemotherapeutic used for cancer treatment. Its use associates with peripheral neuropathies and chronic gastrointestinal side-effects. Oxaliplatin induces immunogenic cell death by provoking the presentation of damage associated molecular patterns. The damage associated molecular patterns high-mobility group box 1 (HMGB1) protein exerts pro-inflammatory cytokine-like activity and binds to toll-like receptors (namely TLR4). Gastrointestinal microbiota may influence chemotherapeutic efficacy and contribute to local and systemic inflammation. We studied effects of oxaliplatin treatment on 1) TLR4 and high-mobility group box 1 expression within the colon; 2) gastrointestinal microbiota composition; 3) inflammation within the colon; 4) changes in Peyer’s patches and mesenteric lymph nodes immune populations in mice. TLR4^+^ cells displayed pseudopodia-like extensions characteristic of antigen sampling co-localised with high-mobility group box 1 -overexpressing cells in the colonic lamina propria from oxaliplatin-treated animals. Oxaliplatin treatment caused significant reduction in *Parabacteroides* and *Prevotella*_*1*_, but increase in *Prevotella*_*2*_ and *Odoribacter* bacteria at the genus level. Downregulation of pro-inflammatory cytokines and chemokines in colon samples, a reduction in macrophages and dendritic cells in mesenteric lymph nodes were found after oxaliplatin treatment. In conclusion, oxaliplatin treatment caused morphological changes in TLR4+ cells, increase in gram-negative microbiota and enhanced HMGB1 expression associated with immunosuppression in the colon.

## Introduction

Platinum-based chemotherapeutic agents are widely used for the treatment of cancer, and oxaliplatin, the third generation drug, is primarily used as the first-line treatment for colorectal malignancies [1,2]. Platinum-based drugs mediate their cytotoxic effects via the formation of nuclear and mitochondrial DNA platinum adducts which ultimately affect cell viability and hinder prospective replication [3–5]. Despite its therapeutic efficacy, the use of oxaliplatin causes unfavourable side-effects which include, but are not limited to, peripheral sensory neuropathy and gastrointestinal dysfunction [2,6–9]. These side-effects are major hurdles for cancer treatment as they result in dose reductions, treatment non-compliance and cessation [7,10,11]. Whilst the peripheral sensory neuropathy caused by oxaliplatin has attracted a large research focus, there are limited studies investigating the effects of this drug on gastrointestinal dysfunction. Only recently, the enteric nervous system (ENS) has gained attention regarding its role in the multifaceted pathophysiology of gastrointestinal dysfunction following chemotherapeutics [8,9,12]. The ENS is an intrinsic and intricate neuronal network embedded throughout the gastrointestinal tract which regulates secretion, absorption, vasomotor tone and motility [13]. The ENS can anatomically be divided into two major plexuses; the submucosal and myenteric. A few studies to date have shown that oxaliplatin induces myenteric neuronal loss, changes in the proportion of neuronal phenotypes, oxidative stress and causes changes in gastrointestinal transit and motility leading to constipation [8,9,14]. However, the mechanisms underlying oxaliplatin-induced changes in the myenteric plexus and cell death remain to be elucidated.

It is well established that anti-cancer agents induce damage to the gastrointestinal mucosa which may cause dysbiosis of commensal microbiota and potentiate inflammation [15–19]. A number of studies have reported microbiota dysbiosis following the anti-cancer chemotherapeutic agents, irinotecan and 5-fluorouracil [20–23]. However, the effects of platinum-based drugs on gastrointestinal microbiota remain largely unexplored. Gastrointestinal inflammation has been associated with persistent alterations in enteric neuron function and neuronal loss [24–26]. The gastrointestinal tract in particular is equipped with lymphoid organs (Peyer’s patches (PPs) and mesenteric lymph nodes (MLNs)) which houses ~70% of the body’s immunocytes, thus, highlighting their important role in discriminating between innocuous and noxious pathogens or danger signals [27]. Further adding to this complexity, oxaliplatin is regarded as a potent inducer of immunogenic cell death. Apoptosis has long been considered to be an immunologically silent or tolerogenic event, however, oxaliplatin treatment has been shown to induce beneficial anti-cancer immune responses through the induction of damage-associated molecular patterns (DAMPs) in colorectal cancer cells [28]. The presentation of DAMPs can prompt the engulfment of dying cells by phagocytes, or apoptotic antigens may be presented to T cells for targeted elimination [28,29]. A classical DAMP is the nuclear-resident non-histone protein high mobility group box 1 (HMGB1) which exerts pro-inflammatory cytokine-like activity once cytoplasmically translocated and/or released into the extracellular environment by damaged cells [28,29]. HMGB1 is a ligand for toll-like receptors and is presented to T cells for priming and activation [29,30]. Both DAMPs and pathogen-associated molecular patterns (PAMPs–microbial endotoxins) can induce an immunological response following anti-cancer chemotherapy [31,32]. It is unknown whether gastrointestinal inflammation may be implicated in enteric neuropathy associated with oxaliplatin treatment, either directly or inadvertently.

Herein, we determined the effects of oxaliplatin treatment on 1) TLR4 and HMGB1 expression within the murine colon; 2) gastrointestinal microbiota composition; 3) inflammation within the colon; and 4) changes in immune populations within the murine PPs and MLNs. It is hypothesized that oxaliplatin treatment will cause gastrointestinal inflammation characterized by an increase in TLR4^+^ and CD45^+^ cells within the colon lamina propria and at the level of the myenteric plexus, will cause changes to microbiota composition, and will induce changes in immune populations within the PPs and MLNs which may contribute to inflammatory enteric neuropathy.

## Materials and methods

### Animals

Male BALB/c mice (n = 40, aged 7–8 weeks, weighing 18-25g) were used in this study. We have previously established an orthotopic mouse model of colorectal cancer in Balb/c mice (35). As cancer and chemotherapy can both differentially affect immune responses, our first aim is to study them exclusive to one another. We have published several papers on the effects of chemotherapy on the enteric nervous system using Balb/c mouse models (8, 9, 14). Our prospective studies are to combine colorectal cancer and chemotherapy. Therefore, Balb/c mouse strain is used in this study. We have based our studies on male mice as fluctuations in hormones during the estrous cycle in female mice may present as confounding factors influencing immune responses.

Mice had access to food and water *ad libitum* and were kept under a 12 hour light/dark cycle in a well-ventilated room at a temperature of 22°C. Mice acclimatized for up to 1 week prior to the commencement of *in vivo* intraperitoneal injections. All efforts were made to minimise animal suffering, to reduce the number of animals used, and to utilise alternatives to *in vivo* techniques, if available. All procedures in this study were approved by the Victoria University Animal Experimentation Ethics Committee (Animal Ethics number 15–011) and performed in accordance with the guidelines of the National Health and Medical Research Council Australian Code of Practice for the Care and Use of Animals for Scientific Purposes.

### Oxaliplatin treatment

Mice were separated into 2 cohorts (n = 4-10/group): 1) vehicle-treated (sterile water), 2) oxaliplatin-treated (3 mg/kg, Sigma-Aldrich, Australia). All mice received intraperitoneal injections (volume calculated per body weight, maximum of 200 μl/injection) using 26 gauge needles tri-weekly totaling 6 injections. Dosages were calculated per body mass as previously published [33,34]. Mice were culled via cervical dislocation 14 days subsequent to their first intraperitoneal injection, and the colon, PPs, and MLNs were harvested.

### Immunohistochemistry

The colon was harvested (n = 4/group), cut along the mesenteric border and pinned to silicone-based petri dishes containing 1x phosphate buffered solution (PBS). Tissues were incubated in Zamboni’s fixative (2% formaldehyde, 0.2% picric acid and 0.1 M sodium phosphate buffer (pH 7.0)) overnight at 4°C. The following day, tissues were washed 3 x 10 minutes in 100% DMSO, followed by 3 x 10 minute washes with 1x PBS. Sections (30μm) were cut and incubated with a mouse blocking reagent (M.O.M. kit, Vector Labs, USA), or 10% normal donkey serum for 1 hour at room temperature, then washed 3 x 10 minutes with PBS and Triton-x100 (PBS-T). Sections were co-labelled with anti-TLR4 (mouse, 1:500, Abcam, USA) and anti-HMGB1 (rabbit, c-terminus, 1:500, Abcam, USA) antibodies or with anti-CD45 (mouse, 1:500, Abcam, USA) antibody alone. Primary antibodies were incubated at room temperature overnight, and were then washed 3 x 10 minutes with PBS-T. The secondary antibody for both TLR4 and CD45 was FITC-conjugated (mouse, 1:200, Abcam, USA) made up in M.O.M. diluents; and the secondary antibody for HMGB1 was AlexaFluor-647-conjugated (rabbit, 1:200, Jackson Immuno-Research, USA). Secondary antibodies were incubated at room temperature for 2 hours and then washed 3 x 10 minutes with PBS-T. Sections were mounted onto glass slides using an anti-fade mounting medium (DAKO, Australia).

### Imaging and analysis

Three-dimensional (z-series) images of the colon cross sections were taken using an *Eclipse Ti* confocal microscope (Nikon, Japan). Excitation wavelengths were set to 473 nm for FITC and 640.4 nm for Alexa Fluor 647. The number of CD45^+^ cells was counted from 8 images/preparation taken at 20x magnification with a total area of 2 mm^2^. CD45^+^ immunoreactivity was measured from 8 images/preparation taken at 20x magnification with a total area of 2 mm^2^. All images were captured under identical conditions, calibrated to standardise minimum baseline fluorescence, and were converted to binary. Differences in fluorescence from baseline were measured using Image J software (National Institute of Health, USA). All images were coded and analyzed blindly.

### Fecal DNA isolation

Fecal pellets were collected from vehicle- and oxaliplatin-treated animals (n = 10/group), and stored at -80°C until time of processing. The PowerFecal DNA Isolation Kit (MO BIO Laboraties Inc, Australia) was used to obtain DNA from the fecal pellets as per manufacturer’s instructions. The isolated fecal DNA was frozen in -80°C until time of high-throughput sequence analysis.

### PCR amplification of variable regions 3–4 of the 16S rRNA

PCR amplification of variable regions 3–4 (V3–V4) from the 16S rRNA gene and subsequent Illumina sequencing were performed commercially by the Australian Genome Research Facility (Brisbane, Australia). The 2-stage PCR protocol was followed according to the Illumina 16S guidelines (http://sapac.support.illumina.com/downloads/16s_metagenomic_sequencing_library_preparation.html). Briefly, PCR amplicons were generated using a Forward 341F primer (CCTAYGGGRBGCASCAG) and a Reverse 806 R primer (GGACTACNNGGGTATCTAAT) along with KAPA HiFi HotStart ReadyMix (Roche, Australia). Thermal cycling consisted of 3 min at 95°C, and 25 cycles consisting of 30 s at 95°C, 30 s at 55°C, and 30 s at 72°C, followed by 5 min at 72°C. Second stage PCR was completed by using Nextera XT Index Kit (Illumina, Australia). Thermal cycling consisted of 3 min at 95°C, and 8 cycles consisting of 30 s at 95°C, 30 s at 55°C, and 30 s at 72°C, followed by 5 min at 72°C. Resulting amplicons were measured by fluorometry, normalised and then pooled with unique indices. This amplicon pool was then run on the Illumina MiSeq platform.

### High-throughput sequence analysis of fecal microbiota

Fecal DNA samples underwent high-throughput sequencing on the Illumina MiSeq platform at the Australian Genome Research facility (University of Queensland, Brisbane, Australia). Paired-end reads were assembled by aligning the forward and reverse reads using join_paired_ends.py. Primers were trimmed using Seqtk (version 1.0). Trimmed sequences were processed using Quantitative Insights into Microbial Ecology (QIIME 1.9) 4 USEARCH2,3 (version 8.0.1623) software (Caporaso et al. 2010). Briefly, de-multiplexing and quality filtering were performed using the split_libraries_fastq.py script for each data set. Operational Taxonomic Units (OTUs) were *de novo* picked at 97% sequence similarity following the usearch pipeline and representative sequences of each cluster were used to assign taxonomy through matching against the Blast 2.2.22 database. Evaluations present at each taxonomic level, including percentage compilations, represent all sequences resolved to their primary identification or their closest relative. Alpha diversity using alpha_diversity.py script was performed for species richness, Good’s coverage, Chao1, Shannon-Wiener’s diversity index and Simpson’s index of diversity. Weighted and unweighted UniFrac distance matrices were obtained through Jack-knifed beta diversities in QIIME and principal coordinate analysis (PCoA) plots were obtained. Sample clustering and statistical analysis were carried out in R environment and SPSS (version 23).

### Myeloperoxidase activity

Colon tissues from vehicle- and oxaliplatin-treated animals (n = 3/group) were harvested and homogenized in 4 volumes of MPO assay buffer using a FastPrep24TM5G homogenizer and matrix D lysing tubes (MP Biomedicals, Australia) for 40 seconds. The supernatant was transferred into collecting tubes, and centrifuged at 13,000 x *g* for 10 minutes at 4°C. Tissue protein levels were quantified using the bicinchoninic acid (BCA) assay (Thermo Scientific, Australia) according to manufacturer’s instructions and absorbance was read at 526 nm using a VarioskanTM Flash Multimode Reader (Thermo Scientific, Australia) using SkanIt software v.2.4.3. The MPO Colorimetric Activity Assay (Sigma-Aldrich, Australia) protocol was followed as per manufacturer’s instructions. Briefly, TNB standards, MPO positive and negative controls and each sample was assayed in triplicate. Samples were normalized to protein content. Absorbance was read at 412 nm using a VarioskanTM Flash Multimode Reader (Thermo Scientific, Australia) using SkanIt software v.2.4.3. All standards and samples were corrected for background absorbance readings. A standard curve plotted and the amount of TNB consumed by the enzyme assay for each sample was determined. MPO activity was calculated as per manufacturer’s instructions: MPO Activity = B Sample Dilution Factor/(Reaction Time) x V. MPO activity is reported as nmol/min/mL = milliunit.

### RNA isolation and RT^2^ profiler PCR arrays

Colons from vehicle (n = 5) or oxaliplatin-treated (n = 4) mice were removed, snap frozen in liquid nitrogen and stored in -80°C until used. Total RNA was extracted using TRIzol (Invitrogen, Carlsbad, California) and further purified using an RNeasy Mini kit (Qiagen, Hilden, Germany), including the on-column DNase digestion step. RNA integrity was determined with an Agilent 2100 Bioanalyzer (Agilent Technologies, USA) using RNA 6000 Nano chips (Agilent Technologies); RNA Integrity Numbers (RIN) of all colon RNA samples were within an appropriate range (vehicle treated: 8.8 ± 0.9, n = 5; oxaliplatin-treated: 9.2 ±0.1, n = 4). Total RNA concentration was determined on a Qubit Fluorometer (Invitrogen) using a Qubit RNA BR Assay. Gene expression was investigated using the pathway specific RT^2^ Profiler PCR Array ‘Mouse Cancer Inflammation and Immunity Crosstalk’ (Qiagen, Cat. no. PAMM-181Z) according to the manufacturer’s instructions. Arrays were performed using equal quantities of either vehicle or oxaliplatin-treated RNA. Reverse transcription was carried out with the RT^2^ First Strand Kit (Qiagen) using 0.5μg pooled RNA as template. Equal amounts of cDNA were distributed to each well of the RT^2^ Profiler Array and real-time PCR was performed in a Biorad CFX96 real-time thermal cycler, using RT^2^ SYBR Green qPCR Mastermix (Qiagen). PCR cycling comprised an initial denaturation step at 95°C for 10 minutes followed by 40 cycles of 95°C for 15 seconds and 60°C for 1 minute. Melt curve analysis was performed to verify PCR specificity. Arrays were performed in duplicate for each RNA pool. C_T_ values (cycle number at which fluorescence crosses a defined threshold) were obtained using the Bio-rad CFX Manager software, using a constant value across all arrays. The detection limit was set at C_T_ of 35 cycles. C_T_ values were uploaded to and analysed using the web-based Gene Globe Data Analysis Center (Qiagen). Data were normalised to the mean of five reference genes: Glyceraldehyde-3-phosphate dehydrogenase, *Gapdh*; Beta-2 microglobulin, *B2m*; Actin-beta, *Actb*; Glucuronidase-beta, *Gusb* and Heat shock protein 90 alpha (cytosolic), class B member 1, *Hsp90ab1*. Fold change was calculated using the ΔΔC_T_ method, as the ratio of normalised gene expression in the oxaliplatin-treated group to normalised gene expression in the vehicle-treated control group.

### Flow cytometry

To identify immune cell changes following oxaliplatin treatment the PPs and MLNs were harvested. An n = 5 PPs or MLNs were collected from each animal. PPs and MLNs were placed in 15 mL tubes containing FACS buffer (PBS, 0.1% bovine serum albumin and 0.02% sodium azide) and were kept on ice. Mucosal epithelium lining the PPs and excess adipose tissue attached to MLNs were gently removed using forceps. Manual cell suspensions of the PPs, MLNs and spleen were performed. All samples were then centrifuged at 1500 rpm for 5 minutes at 4°C. The supernatant of each cell suspension was aspirated and the pellet containing the immune cells was then resuspended in 1 mL of FACS buffer and filtered. Manual cell counts were performed, and cells were seeded to 96 U-bottom well plates (BD Biosciences, USA) and centrifuged at 1300 rpm for 3 minutes at 4°C. Subsequent to centrifugation, the 96 U-bottom well plates were then aspirated. A selection of cell surface antibodies was used to identify various immune cell populations **(Table 1)**. Each antibody cocktail was loaded to appropriate wells, and incubated for 20 minutes at 4°C. Subsequent to the incubation period, the cells were washed with FACS buffer and centrifuged at 1300rpm for 3 minutes at 4°C. The plates were aspirated and cells within each well were resuspended in FACS buffer, and then transferred to FACS tubes. BD Biosciences LSR II and FACS CANTO II flow cytometers were used to collect 200,000 cells from each cell suspension. Information was obtained via software FACSDiva (BD Biosciences, USA), and analysis was conducted using FlowJo (Tree Star, USA) or FACSDiva.

**Table 1:** Antibodies used for FACS experiments.

Cells	Primary antibody	Conjugate	Host species	Dilution
**Pan-leukocyte marker**	CD45	PerCP/Cy5.5	Mouse	1:400
**Pan-T cell marker**	CD3	PerCP/Cy5.5	Mouse	1:400
**T cell receptor**	TCRβ	APC	Rat	1:250
**Granulocytes**	GR-1, CD11b	PE-Cy7	Rat	1:100
**Cytotoxic T cells**	CD8	Pacific Blue	Rat	1:100
**Helper T cells**	CD4	Pacific Orange	Rat	1:100
**B cells**	B220	FITC	Mouse	1:400
**Macrophages**	CD11b, Ly6C, Ly6G, CD206, F4/80	PE	Rat	1:200
**Dendritic cells**	CD11c	Pacific Blue	Rat	1:250
**Major histocompatibility complex II**	MHC-II	Brilliant Violet 510	Rat	1:800
**Eosinophils**	CD193	Alexa Fluor 647	Rat	1:200
**NK cells**	CD49b	PE	Rat	1:100
**γδ T cells**	γδ-TCR	FITC	Mouse	1:500
**NKT cells**	CD1d α-Galactosylceramide tetramer	PE	Rat	1:500

### Statistical analysis

For microbiota studies, two-tailed *t*-tests were used to compare two sets of data, assuming unequal variance. The data generated by mass spectral analyses were normalized with respect to internal standards (RSD = 19.28%), where a magnitude of 1 fold change referred to the concentration of 10 mg/L. Statistical analysis was performed using SIMCA 14 (Umetrics AG, Umeå, Sweden). Statistical analysis for all other experiments included an unpaired *t*-test with Welch’s correction using GraphPad PrsimTM v6.0 (GraphPad Software, USA). The data were represented as mean ± standard error of the mean (SEM). Statistical significance for all experiments was defined where the *P* value was less than 0.05.

## Results

### Animal symptoms

During the course of oxaliplatin treatment, mice failed to gain weight compared to their control counterparts, and display signs of nausea (pica), as well as constipation.

### Oxaliplatin treatment causes morphological changes in TLR4^+^ cells and reduces TLR7, TLR9 and H2-D1 expression in the colon

**Fig 1:**
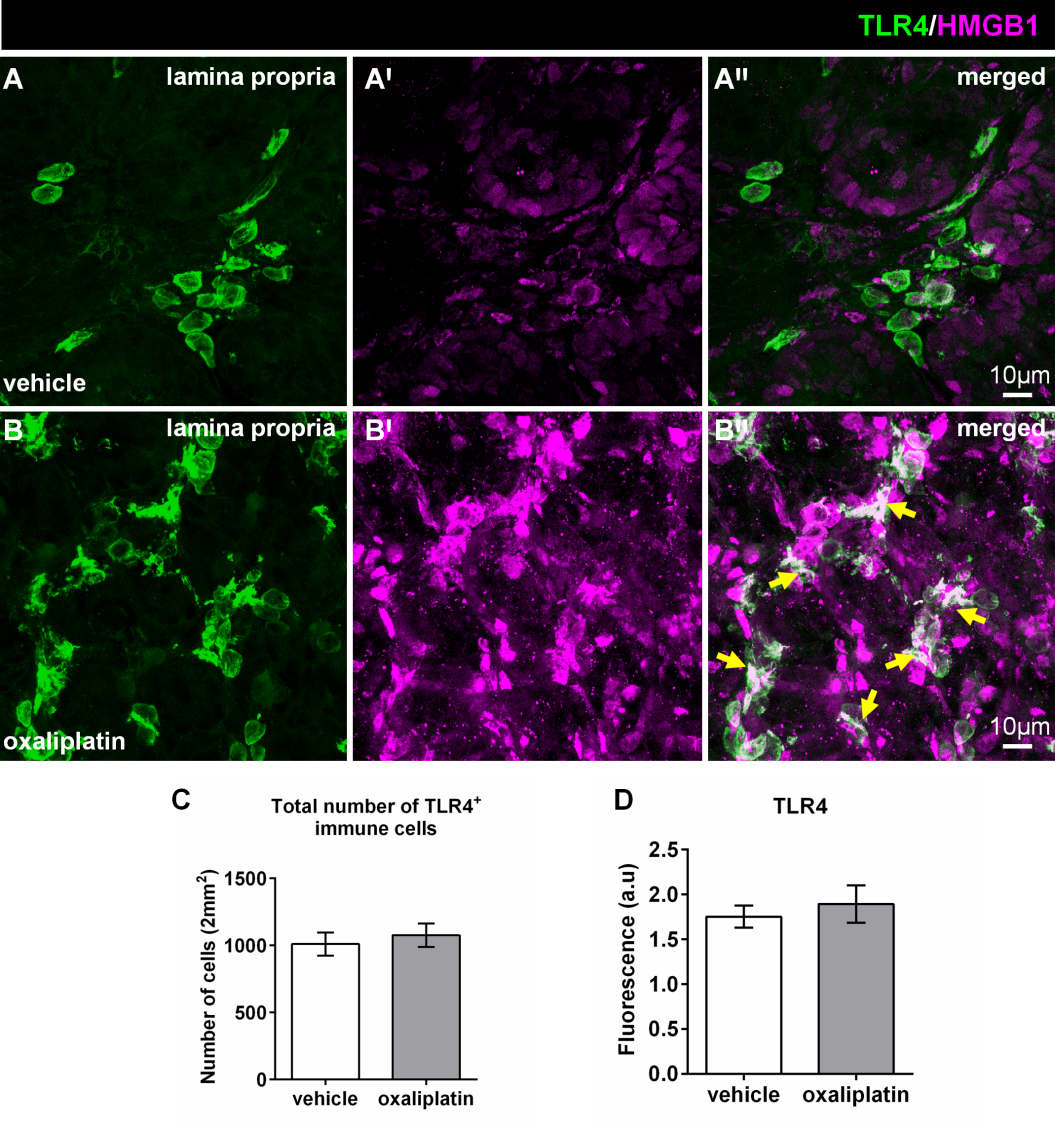
Effects of oxaliplatin treatment on HMGB1 expression and co-localisation with TLR4 in the lamina propria of the colon. Colon cross-sections (30μm thick) from the vehicle and oxaliplatin-treated groups were labelled with anti-TLR4 (green) and anti-HMGB1 (magenta) antibodies **(A-B′′)**. Strong HMGB1 immunoreactivity is observed within the lamina propria **(B′)** of the colon from the oxaliplatin-treated animals when compared to the vehicle-treated cohort **(A′).** Greater co-localisation of TLR4 and HMGB1 is evident within the lamina propria **(B′′)** following oxaliplatin treatment when compared to the vehicle-treated cohort **(A′′)**. The numbers of TLR4+ cells were counted from 8 images per preparation taken at 20x magnification with a total area of 2mm^2^. No differences in the total number **(C),** or TLR4+ immunoreactivity **(D)** was observed following oxaliplatin treatment when compared to the vehicle group. Scale = 10μm; n = 4-5/group.

Colon cross-sections from the vehicle and oxaliplatin-treated groups were double-labelled with anti-TLR4 and anti-HMGB1 antibodies to determine any expressional changes. Intense HMGB1 expression within the lamina propria of the colon from the oxaliplatin-treated animals was noted when compared to the vehicle-treated cohort **(Fig 1A–1B′′)**. Furthermore, co-localization of TLR4 and HMGB1 was observed in the lamina propria following oxaliplatin treatment when compared to the vehicle-treated cohort **(Fig 1A–1B′′;** yellow arrows). There were no differences in the total number of TLR4^+^ cells between the vehicle-treated (1010 ± 85) and oxaliplatin-treated groups (1077 ± 87), n = 4-5/group **(Fig 1C)**. Furthermore, no changes in the total TLR4^+^-immunoreactivity amongst vehicle-treated (1.7 ± 0.1) and oxaliplatin-treated cohort (1.8 ± 0.2), n = 4-5/group **(Fig 1D)** were demonstrated.

**Fig 2:**
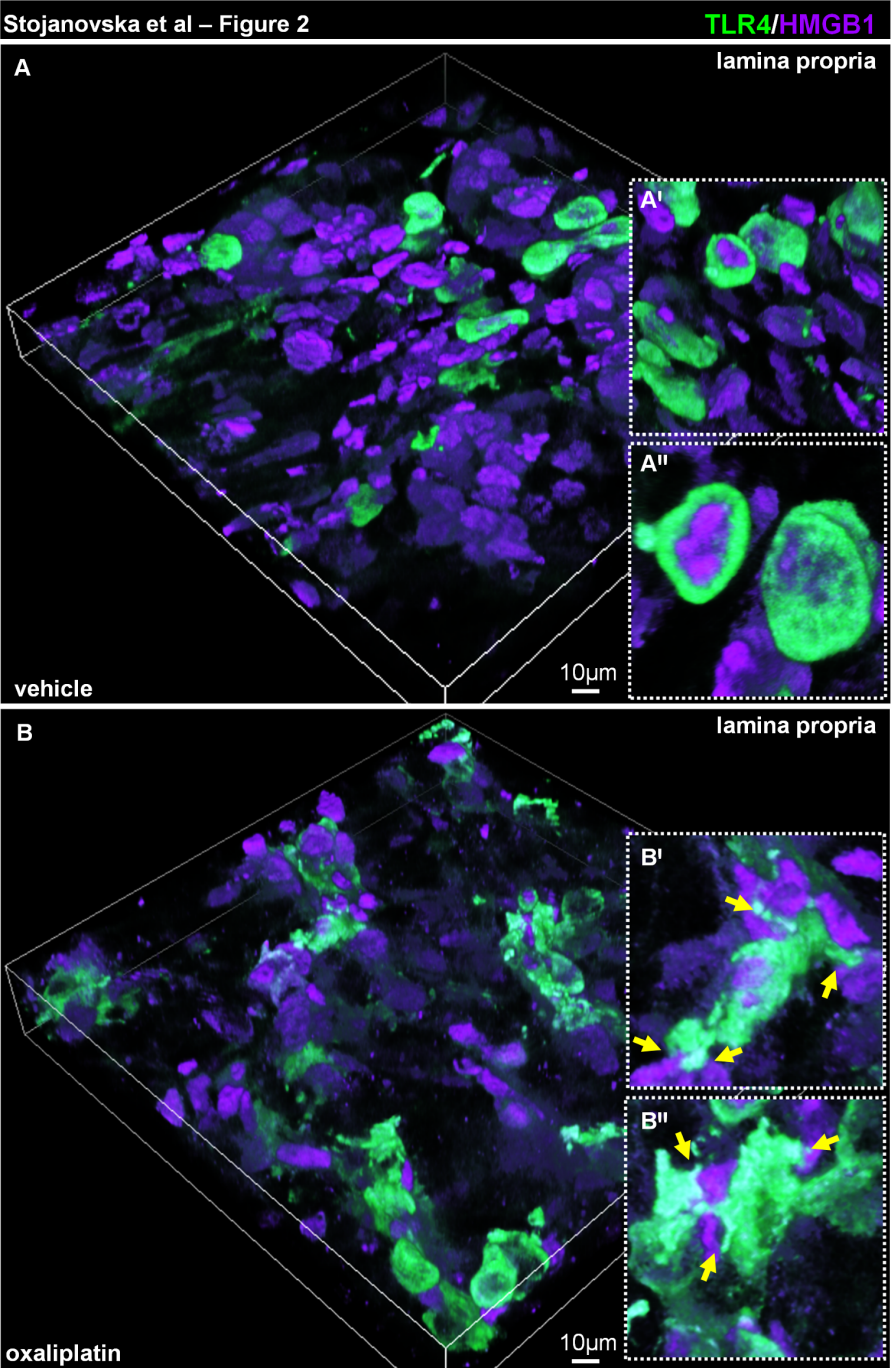
Changes in TLR4^+^ cell morphology and interaction with HMGB1 in the lamina propria of the colon following oxaliplatin treatment. Colon cross-sections (30μm thick) from the vehicle **(A)** and oxaliplatin-treated **(B)** mice were labelled with anti-TLR4 (green) and anti-HMGB1 (magenta) antibodies and presented as 3D reconstruction of confocal z-series slices. TLR4^+^ cells within the lamina propria of the colon from the oxaliplatin-treated animals form contacts with HMGB1 through extending processes **(B′-B′′,** insets with yellow arrows), as opposed to the spherical morphology of TLR4^+^ cells in the vehicle-treated cohort **(A′-A′′,** insets), n = 4/group.

TLR4^+^ cells within the vehicle-treated cohort display minimal contact/co-localisation with HMGB1 **(Fig 2A–2A′′)**. Whereas, TLR4^+^ cells co-localising with HMGB1 within the lamina proporia from oxaliplatin-treated group displayed pseudopodia-like extensions characteristic of antigen sampling **(Fig 2B–2B′′)**.

**Fig 3:**
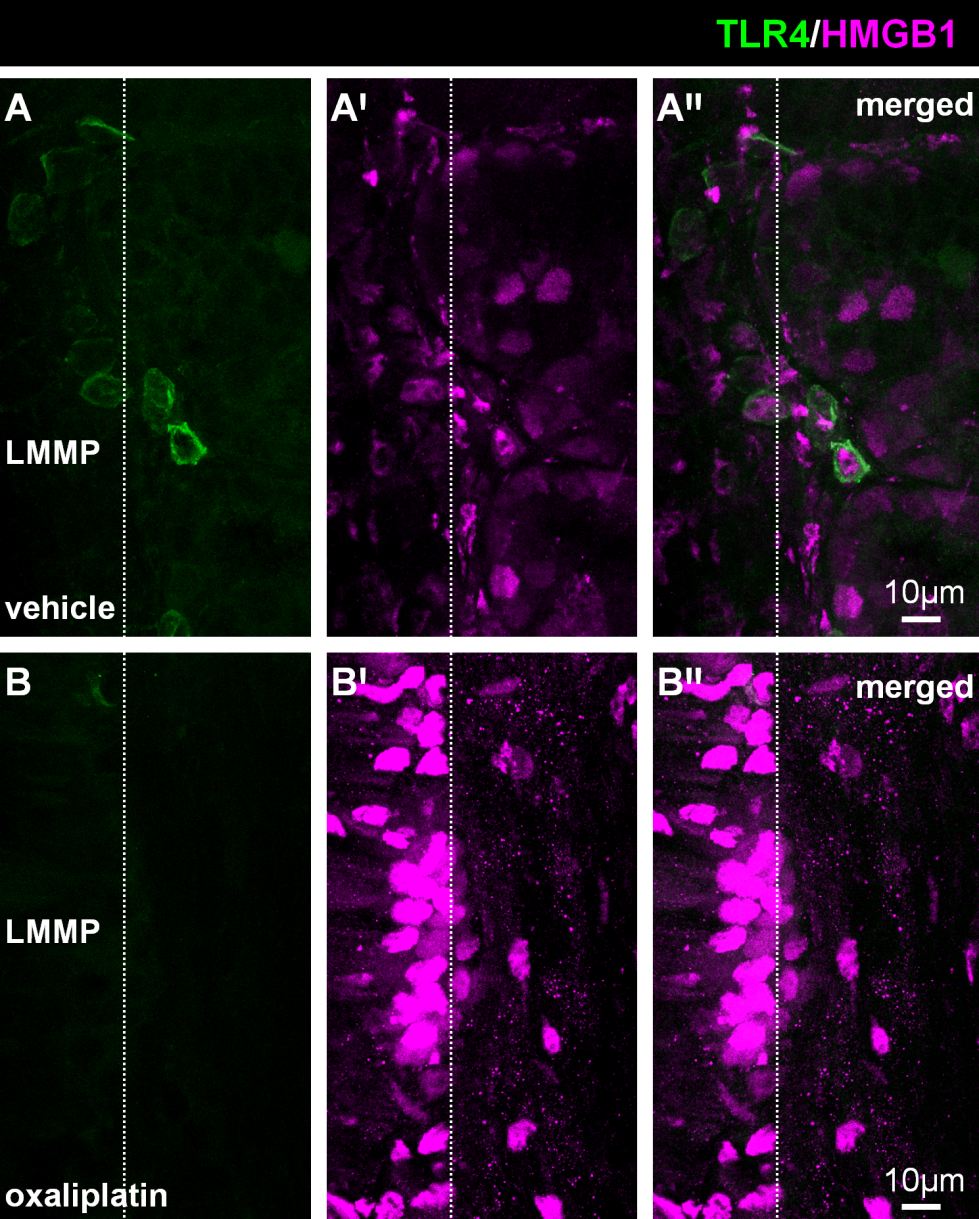
Effects of oxaliplatin treatment on HMGB1 expression and co-localisation with TLR4 in the longitudinal muscle-myenteric plexus (LMMP) of colon. Colon cross-sections (30μm thick) from the vehicle and oxaliplatin-treated groups were labelled with anti-TLR4 (green) and anti-HMGB1 (magenta) antibodies **(A-B′′)**. No TLR4^+^ cells infiltrated the level of the LMMP in either group **(A, B)**.Strong HMGB1 immunoreactivity is observed within the colon from the oxaliplatin-treated animals **(B′)** when compared to the vehicle-treated cohort **(A′).** Scale = 10μm; n = 4/group.

Similar to the lamina propria, there was strong HMGB1 immunoreactivity within the LMMP of the colon from the oxaliplatin-treated group when compared to the vehicle-treated cohort **(Fig 3A–3B′′)**. However, no TLR4^+^ cells infiltrated the LMMP layer of the colon following either treatment (white arrow; n = 4/group).

**Fig 4:**
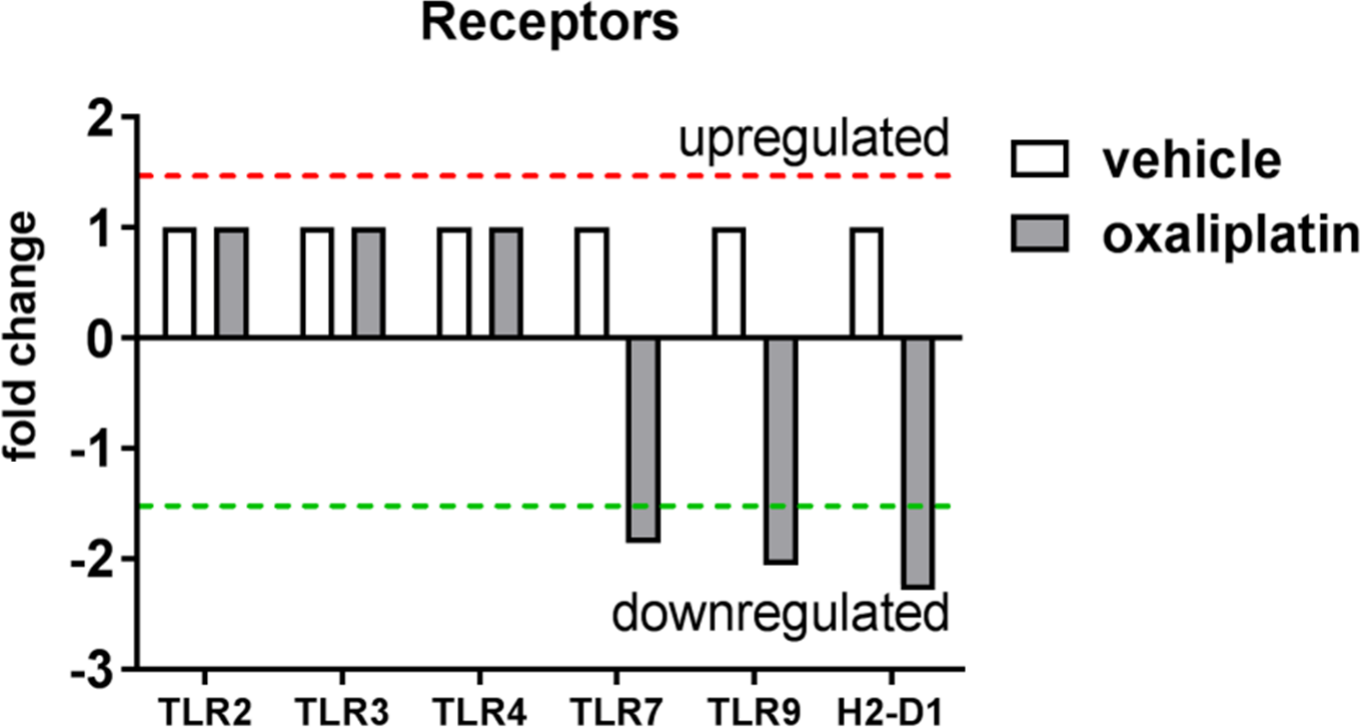
Effects of oxaliplatin treatment on mRNA expression immune receptors. To determine whether oxaliplatin treatment induced changes in the expression of receptors within the colon, RT^2^ Profiler PCR arrays were performed using pooled RNA samples from (vehicle, n = 5; oxaliplatin, n = 4) samples. Oxaliplatin treatment caused a down-regulation of TLR7, TLR9 and H2-D1 mRNA expression when compared to the vehicle-treated cohort. No change in TLR2, TLR3 or TLR4 expression was observed following oxaliplatin treatment.

Interestingly, oxaliplatin treatment induced down-regulation of TLR7 mRNA expression (-1.81 fold change) and TLR9 (-2.01 fold change) when compared to the vehicle-treated group **(Fig 4)**. No changes in TLR2, TLR3 or TLR4 expression were noted following oxaliplatin treatment **(Fig 4)**. In addition, oxaliplatin treatment was associated with reduced expression of Histocompatibility 2, D region locus 1 (H2-D1; -2.23 fold change) when compared to the vehicle-treated group, however H2-D1 mRNA expression was low in both groups (C_T_ ≥ 33 cycles).

### Oxaliplatin treatment has no effect on richness, diversity and evenness of intestinal microbiota, but causes changes at the genus level

To compare compositional differences in the gut microbiota amongst vehicle-treated and oxaliplatin-treated mice, 16S rRNA sequencing was conducted. Total DNA was isolated from fecal samples (n = 10 mice/group), and PCR amplicons spanning the 16S rRNA V3-V5 hypervariable region were sequenced. Microbiota composition was assessed with regards to operational taxonomic units (OTUs) Chao richness, Shannon diversity index, Simpson’s diversity index, as well as unweighted UniFrac Principal Coordinate Analysis (PCoA).

**Fig 5:**
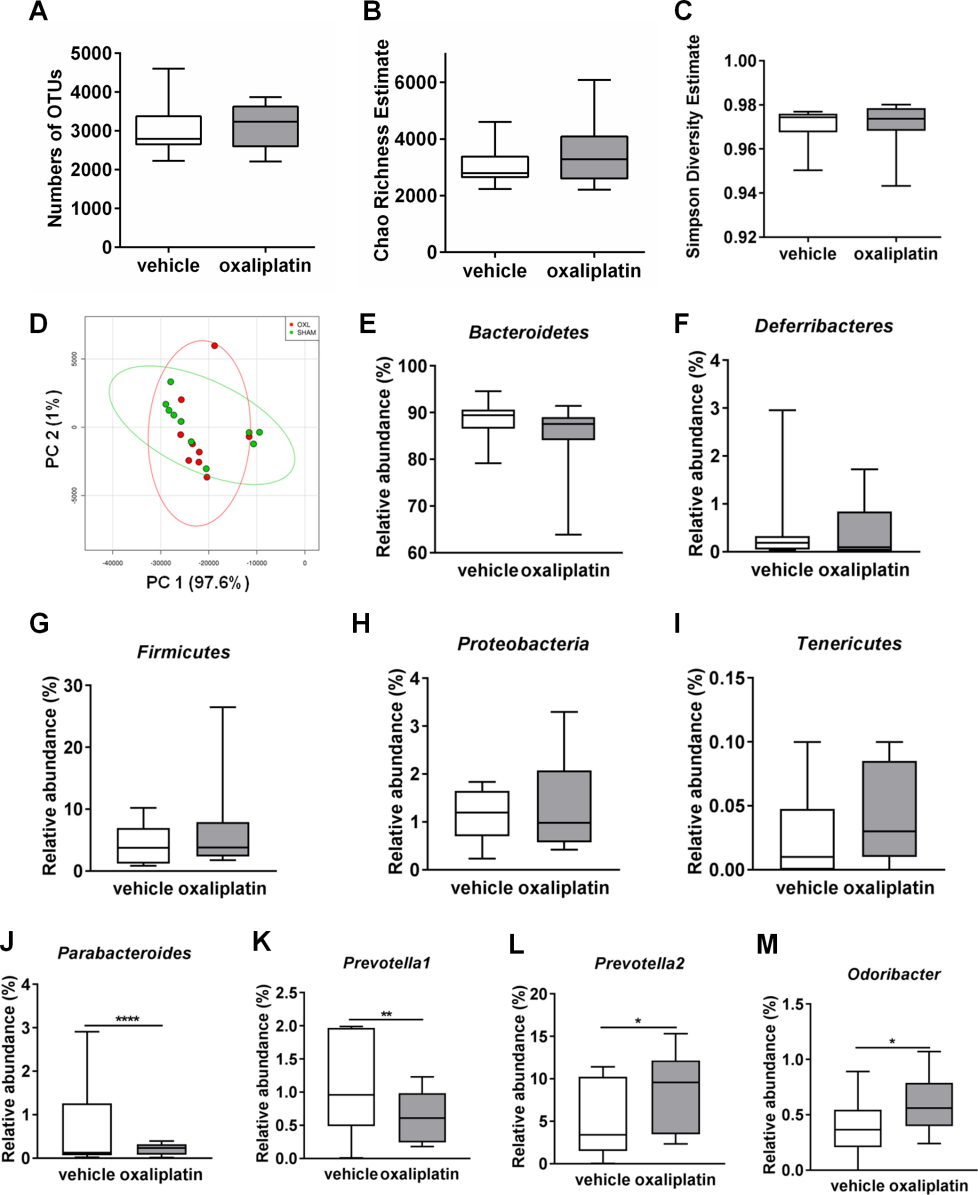
Effects of oxaliplatin treatment on the composition of intestinal microbiota. Fecal microbiota Chao richness estimate, Simpson diversity estimate and evenness following oxaliplatin treatment were analysed using 16S rRNA sequencing. Oxaliplatin treatment did not cause any significant changes to the number of OTUs **(A)**, Chao richness estimate **(B)**, Simpson diversity estimate **(C)** or PCoA unweighted UniFrac distance percentages **(D)**. Oxaliplatin treatment did not cause any significant changes to five dominant phyla groups identified in the fecal microbiota: *Bacteroidetes* **(E)**, *Deferribacteres* **(F)**, *Firmicutes* **(G)**, *Proteobacteria* **(H)** and *Tenericutes* **(I)**. Oxaliplatin treatment caused a significant reduction in *Parabacteroides* (J) and *Prevotella*_*1*_ (K) *species*, and a significant increase in *Prevotella*_*2*_ (L) and *Odoribacter* species **(M)**. *P<0.05; **P<0.01; ****P<0.0001; n = 10/group.

No significant difference in the numbers of OTUs was observed between the vehicle-treated (3058 ± 233.6) and the oxaliplatin-treated mice (3134 ± 175.6) **(Fig 5A)**. Furthermore, no significant difference in Chao richness was observed amongst the vehicle-treated (3058 ± 233.6) and the oxaliplatin-treated cohort (3562 ± 381.5) **(Fig 5B)**. There were no significant differences in diversity between the vehicle-treated (0.97 ± 0.002) and the oxaliplatin-treated group (0.971 ± 0.003) **(Fig 5C)**. Principle coordinate analysis (PCOA) from oxaliplatin-treated mice microbiome based upon unweighted UniFrac distance did not show significant difference when compared to the vehicle-treated cohort **(Fig 5D)**.

In this study, five major phyla groups were identified: *Bacteroidetes*, *Deferribacteres*, *Firmicutes*, *Proteobacteria* and *Tenericutes*. Although *Bacteroidetes* was the most abundant phylum species, no significant differences were observed between the vehicle-treated (88.3 ± 1.2) and oxaliplatin-treated mice (84.9 ± 2.2) **(Fig 5E)**. No significant differences in *Deferribacteres* phyla were observed amongst the vehicle-treated (0.45 ± 0.25) and the oxaliplatin-treated cohort (0.40 ± 0.16) **(Fig 5F)** as well as to *Firmicutes* (1.14 ± 0.15; vehicle treated) compared to (1.35 ± 0.29; oxaliplatin-treated group) **(Fig 5G)**. In addition, there were no significant changes to *Proteobacteria* abundance following oxaliplatin treatment (1.35 ± 0.29) compared to vehicle-treatment (1.14 ± 0.15) **(Fig 5H)**. Furthermore, no changes in *Tenericutes* phyla were observed amongst the vehicle-treated (0.03 ± 0.009) and oxaliplatin-treated mice (0.04 ± 0.01) **(Fig 5I)**.

Changes to the composition of the microbiota genera following oxaliplatin treatment was determined using 16S rRNA sequencing. Twelve common species present in both the vehicle-treated and the oxaliplatin-treated cohort were identified. These species included: *Bacteroides*, *Parabacteroides*, *Prevotella*_*1*_, *Prevotella*_*2*_, *Odoribacter*, *Mucispirillum*, *Lactobacillus*, *Dehalobacterium*, *Ruminococcus*, *Sutterella*, *Bilophila* and *Desulfovibrio*. The particular taxonomy for *Prevotella*^*1*^ and *Prevotella*^*2*^ is yet to be determined. Unknown species *‘unknown’* were also detected in both the vehicle-treated and the oxaliplatin-treated cohorts **(Table 2)**. A significant reduction in *Parabacteroides* (vehicle-treated: 0.71 ± 0.003; oxaliplatin-treated: 0.21 ± 0.04; *P*<0.0001) and *Prevotella*_*1*_ species (vehicle-treated: 1.06 ± 0.002; oxaliplatin-treated: 0.64 ± 0.13; *P*<0.05) was noted in the oxaliplatin-treated group when compared to the vehicle-treated cohort **(Fig 5J and 5K, Table 2)**. Oxaliplatin treatment induced a significant increase in the *Prevotella*_*2*_ species (vehicle-treated: 4.87 ± 0.01; oxaliplatin-treated: 8.58 ± 1.4; *P*<0.05) and in the *Odoribacter* species (vehicle-treated: 0.39 ± 0.0007; oxaliplatin-treated: 0.62 ± 0.08; *P*<0.05) when compared to the vehicle-treated group **(Fig 5L and 5M; Table 2)**. No changes to *Bacteroides*, *Mucispirillum*, *Lactobacillus*, *Dehalobacterium*, *Ruminococcus*, *Sutterella*, *Bilophila*, *Desulfovibrio* or ‘unknown’ species were shown.

**Table 2:** Changes to microbiota at the genus level following oxaliplatin treatment.

	Vehicle			Oxaliplatin		
	Mean	SEM	N	Mean	SEM	N
***Bacteroides***	5.80	0.012	10	5.56	1.54	10
***Parabacteroides***	0.71	0.003	10	**** 0.21	0.04	10
***Prevotella***_***1***_	1.06	0.002	10	** 0.64	0.13	10
***Prevotella***_***2***_	4.87	0.013	10	* 8.58	1.41	10
***odoribacter***	0.39	0.0007	10	* 0.62	0.08	10
***Mucispirillum***	0.45	0.002	10	0.40	0.18	10
***Lactobacillus***	1.01	0.002	10	2.28	0.67	10
***Dehalobacterium***	0.19	0.0004	10	0.17	0.04	10
***Ruminococcus***	0.01	0.00005	10	0.01	0.00	10
***Sutterella***	0.36	0.001	10	0.40	0.07	10
***Bilophila***	0.19	0.0006	10	0.19	0.06	10
***Desulfovibrio***	0.51	0.001	10	0.71	0.28	10
***Unknown***	5.68	0.005	10	6.55	0.52	10

*P<0.05

**P<0.01

****P<0.0001. Mean = % abundance; SEM, standard error of the mean

### Lack of immune responses in the colon following oxaliplatin treatment

**Fig 6:**
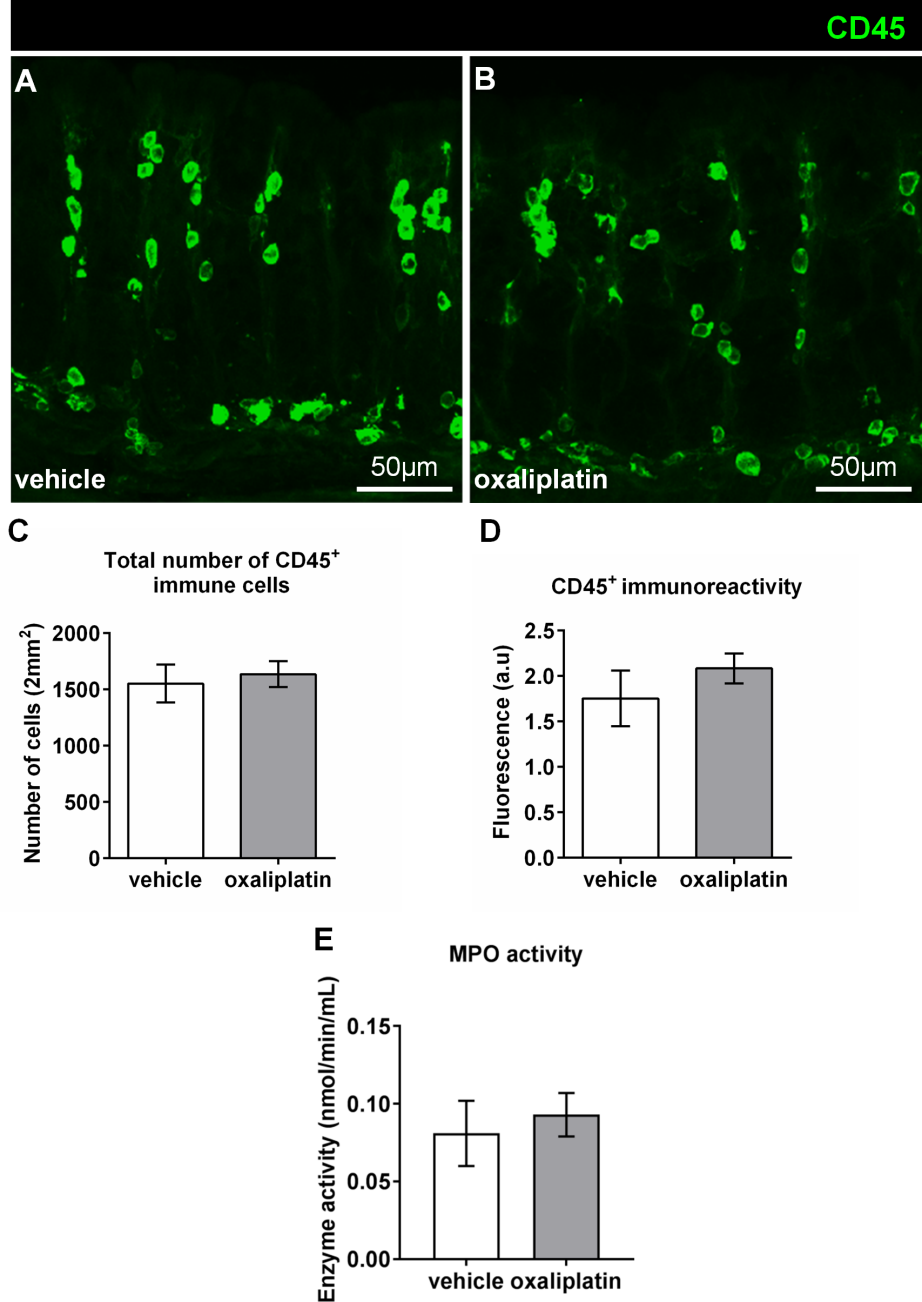
Effects of oxaliplatin treatment on the fluorescence and number of CD45^+^ immune cells, and MPO activity in the colon. Colon cross-sections (30μm thick) from the vehicle and oxaliplatin-treated groups were labelled with the pan-leukocyte marker anti-CD45 antibody (green) **(A-B:** scale bars = 50μm). The numbers of CD45+ cells were counted from 8 images per preparation taken at 20x magnification with a total area of 2mm^2^. No significant differences in the total number of CD45^+^ cells **(C)**, or immunoreactivity **(D)** within the colon is observed between the vehicle-treated and oxaliplatin-treated cohorts, n = 4-5/group. To determine whether oxaliplatin treatment caused inflammation within the colon specific to neutrophils or macrophages a MPO assay was conducted. Oxaliplatin treatment did not induce any significant changes to MPO activity within the colon when compared to the vehicle-treated cohort **(E)**. N = 4/group.

The pan-leukocyte marker anti-CD45 antibody was used to label immunocytes in the colon **(Fig 6A and 6B)**. There were no differences in the total number of CD45^+^ cells between the vehicle-treated (1553 ± 169) and oxaliplatin-treated groups (1666 ± 143), n = 4/group **(Fig 6C)**. No significant changes in CD45^+^ immunoreactivity to indicate inflammation in the colon was observed following oxaliplatin treatment (2.08 ± 0.16) when compared to the vehicle-treated cohort (1.75 ± 0.30), n = 4/group **(Fig 6D)**. Furthermore, no CD45^+^ immune cells were found at the level of the myenteric ganglia. MPO is a peroxidase enzyme with antimicrobial capacity and is typically used as a biomarker for inflammation. No significant difference in MPO activity was shown between the oxaliplatin-treated (0.093 ± 0.01 nmol/min/mL; n = 4) and vehicle-treated groups (0.08 ± 0.02 nmol/min/mL), n = 4/group **(Fig 6E)**.

**Fig 7:**
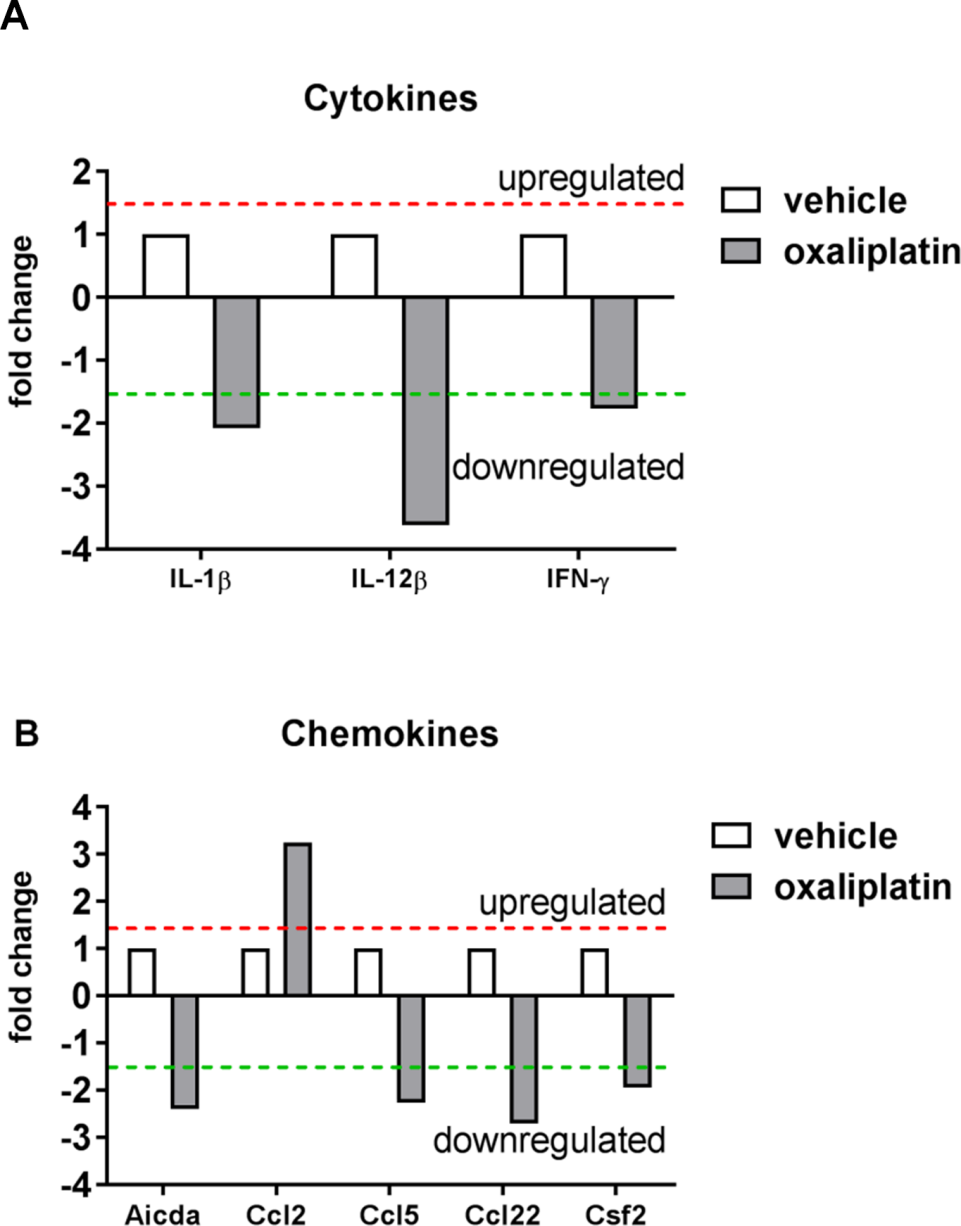
Effects of oxaliplatin treatment on cytokine and chemokine mRNA expression. To determine whether oxaliplatin treatment induced changes in inflammatory mediators within the colon, RT^2^ Profiler PCR arrays were performed using pooled RNA samples from (vehicle, n = 5; oxaliplatin, n = 4) samples. Oxaliplatin treatment caused the down-regulation of the cytokines IL-1β, IL-12β mRNA expression when compared to the vehicle-treated group **(A)**. Oxaliplatin treatment induced the up-regulation of the chemokine Ccl2, and the down-regulation of Ccl5 and Ccl22 chemokine mRNA expression when compared to the vehicle-treated cohort **(B)**.

In order to profile changes in gene expression associated with inflammation, RT^2^ Profiler PCR arrays of colon RNA were performed using pooled RNA samples. Oxaliplatin treatment caused the down-regulation of the cytokines interleukin (IL)-1β (-2.02 fold change) and IL-12β (-3.56 fold change). Reduced interferon gamma mRNA expression (IFN-ᵧ; -1.71 fold change) was also observed following oxaliplatin treatment, however expression was low (C_T_≥34) in both vehicle and treated groups **(Fig 7A)**. Moreover, oxaliplatin treatment caused higher mRNA expression of the chemokine ligand Ccl-2 (3.25 fold change) and lower expression of Ccl5 (-2.19 fold change) and Ccl22 (-2.63 fold change) **(Fig 7B)**. Lower levels of activation induced cytidine deaminase, Aicda; (-2.32 fold change) and colony stimulating factor 2 (Csf2; -1.87 fold change) were also observed following oxaliplatin treatment, however these genes were also expressed at low levels (C_T_≥33) in both vehicle and oxaliplatin-treated samples. Overall most genes on the array showed no change in mRNA expression in the oxaliplatin-treated group, when compared to the vehicle-treated group **(S1 Table).**

### Oxaliplatin treatment induces changes in immune cell populations within the MLNs, but not PPs

**Fig 8:**
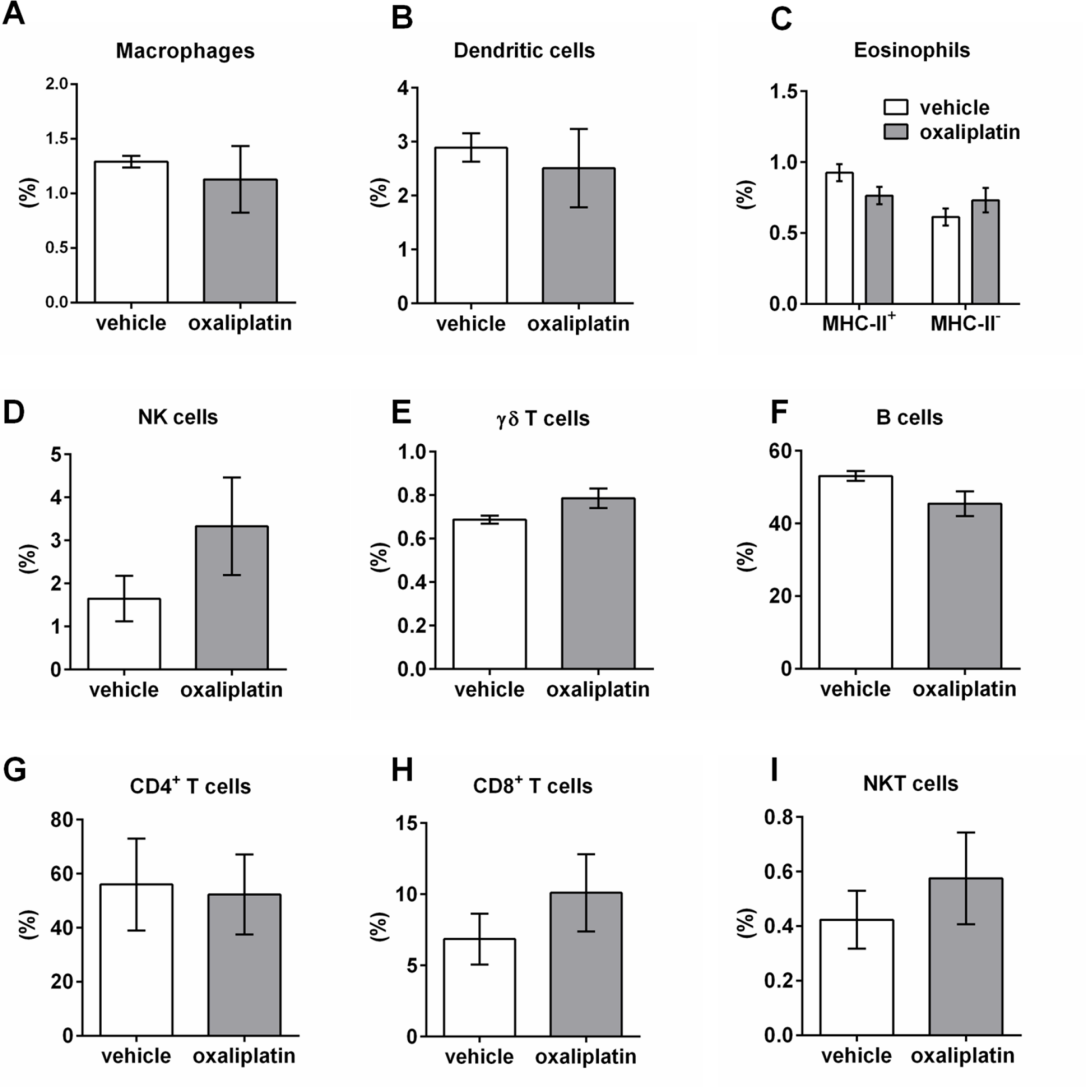
Immune cell populations within the PPs from vehicle and oxaliplatin-treated mice. A series of gating strategies were used to identify immune cell populations within the PPs: macrophages (F4/80^+^ MHC-II^+^); dendritic cells (CD11c^+^ MHC-II^+^); eosinophils (CD11B^+^ MHC-II^-^ Gr-1^+^ CD193^+^ and CD11B^+^ MHC-II^+^ Gr-1^+^ CD193^+^); NK cells (CD49b^+^ TCR^-^); γδ T cells (γδ-TCR^+^ TCRβ^-^); B cells (CD45^+^ TCRβ^-^ B220^+^); CD4^+^ T cells (CD4^+^ TCR^+^); CD8^+^ T cells (CD8^+^ TCR^+^); NKT cells (CD1d α-Galcer tetramer^+^ TCR^+^). No significant differences were observed in any immune cell types within PPs **(A-I)**, n = 5/group.

**Fig 9:**
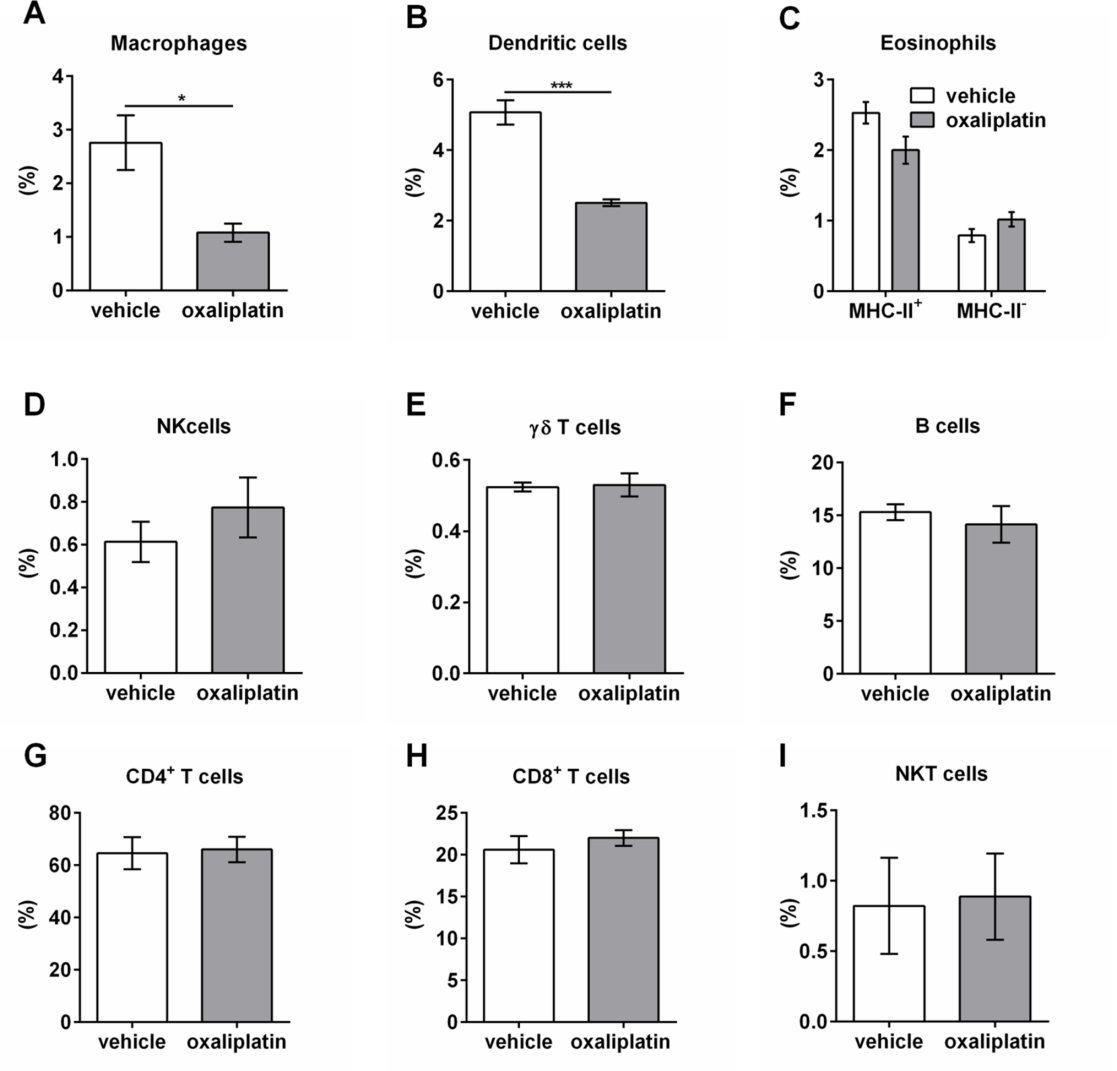
Immune cell populations within the MLNs from vehicle and oxaliplatin-treated mice. A series of gating strategies were used to identify immune cell populations within the MLNs: macrophages (F4/80^+^ MHC-II^+^); dendritic cells (CD11c^+^ MHC-II^+^); eosinophils (CD11B^+^ MHC-II^-^ Gr-1^+^ CD193^+^ and CD11B^+^ MHC-II^+^ Gr-1^+^ CD193^+^); NK cells (CD49b^+^ TCR^-^); γδ T cells (γδ-TCR^+^ TCRβ^-^); B cells (CD45^+^ TCRβ^-^ B220^+^); CD4^+^ T cells (CD4^+^ TCR^+^); CD8^+^ T cells (CD8^+^ TCR^+^); NKT cells (CD1d α-Galcer tetramer^+^ TCR^+^). Oxaliplatin treatment caused a significant reduction in the proportion of macrophages and dendritic cells **(A, B)**, with no effects on eosinophils, NK cells, γδ T cells, B cells (CD45^+^ TCRβ^-^ B220^+^); CD4^+^ T cells (CD4^+^ TCR^+^); CD8^+^ T cells (CD8^+^ TCR^+^); NKT cells (CD1d α-Galcer tetramer^+^ TCR^+^) within MLNs **(C-I)**. *P<0.05, n = 5/group.

To determine the effects of oxaliplatin treatment on the gastrointestinal immune response, we profiled granulocyte and lymphocyte populations within the PPs and MLNs using FACS. Several immune cell populations were gated by the following: macrophages (F4/80^+^ MHC-II^+^); dendritic cells (CD11c^+^ MHC-II^+^); eosinophils (CD11B^+^ MHC-II^-^ Gr-1^+^ CD193^+^ and CD11B^+^ MHC-II^+^ Gr-1^+^ CD193^+^); NK cells (CD49b^+^ TCR^-^); γδ T cells (γδ-TCR^+^ TCRβ^-^); B cells (CD45^+^ TCRβ^-^ B220^+^); CD4^+^ T cells (CD4^+^ TCR^+^); CD8^+^ T cells (CD8^+^ TCR^+^); NKT cells (CD1d α-Galcer tetramer^+^ TCR^.^ Oxaliplatin treatment did not cause any significant changes to the proportion of immune cells within the PPs when compared to the vehicle-treated cohort **(Fig 8A–8I, Table 3**; n = 5/group). However, oxaliplatin treatment resulted in a significant reduction in the proportion of macrophages and dendritic cells within the MLNs when compared to vehicle-treated group **(Fig 9A and 9B, Table 4)**. No changes in other immune cell populations were noted **(Fig 9C–9I**, n = 5/group).

**Table 3:** Proportions of various immune cell populations within the PPs following vehicle and oxaliplatin treatment.

Immune cell population	Vehicle	Oxaliplatin
**Macrophages**	1.3 ± 0.1	1.1 ± 0.3
**Dendritic cells**	2.9 ± 0.3	2.5 ± 0.7
**Eosinophils: MHC-II**^**+**^ **MHC-II-**	0.8 ± 0.2 0.6 ± 0.1	0.7 ± 0.01 0.7 ± 0.1
**NK cells**	1.7 ± 0.5	3.3 ± 1.1
**γδ T cells**	0.7 ± 0.01	0.8 ± 0.04
**B cells**	53.1 ± 1.4	45.5 ± 3.4
**CD4**^**+**^ **T cells**	56.0 ± 17.0	52.3 ± 14.8
**CD8**^**+**^ **T cells**	6.9 ± 1.8	10.1 ± 2.7
**NKT cells**	0.4 ± 0.1	0.6 ± 0.17

**Table 4:** Proportions of various immune cell populations within the MLNs following vehicle and oxaliplatin treatment.

Immune cell population	Vehicle	Oxaliplatin
**Macrophages**	2.8 ± 0.5	*1.1 ± 0.2
**Dendritic cells**	5.1 ± 0.3	***2.5 ± 0.1
**Eosinophils: MHC-II**^**+**^ **MHC-II-**	2.5 ± 0.2 0.8 ± 0.1	2.0 ± 0.2 1.0 ± 0.1
**NK cells**	0.6 ± 0.1	0.8 ± 0.1
**γδ T cells**	0.5 ± 0.01	0.5 ± 0.03
**B cells**	15.3 ± 0.8	14.2 ± 1.7
**CD4**^**+**^ **T cells**	64.6 ± 6.1	66.0 ± 4.8
**CD8**^**+**^ **T cells**	20.6 ± 1.6	22.0 ± 0.9
**NKT cells**	0.8 ± 0.3	0.9 ± 0.3

*P<0.05

***P<0.001.

## Discussion

This study is the first to determine the potential for oxaliplatin treatment to induce inflammatory enteric neuropathy within the murine colon. Oxaliplatin is a potent immunogenic cell death inducer, thus, it was hypothesized that mucosal and neuronal damage would be associated with an inflammatory response [12,28,29,35]. However, in this study we have shown that oxaliplatin treatment does not induce inflammation or changes in total TLR4 immunoreactivity, despite noticeable changes in HMGB1 expression which is a TLR4 ligand. It is well established that cytoplasmic and/or released HMGB1 can exert pro-inflammatory cytokine-like activity capable of inducing strong immunological responses [36,37]. In immunogenic cell death HMGB1 is passively released and acts as a potent ‘eat me’ signal, however, oxidative stress and apoptosis can blunt this pro-inflammatory signalling [38–40]. In this study, HMGB1 and TLR4 co-localisation as well as morphological differences in TLR4^+^ cells within the lamina propria, but not in the LMMP following oxaliplatin treatment were observed. The TLR4^+^ cells co-localised with HMGB1 show pseudopodia-like morphology which is characteristic of antigen sampling [41]. Under conventional circumstances, antigen presenting cells migrate to their nearest draining lymph nodes (such as PPs and MLNs in this case) upon antigen recognition for the priming and activation of T cells. Despite the HMGB1 and TLR4 interaction, there was no amplification of immune cell populations within the lymphoid organs (PPs and MLNs). Presumably, HMGB1 could still be sampled by antigen presenting cells, but may be regarded as an innocuous/neutral molecule. This is apparent when HMGB1 becomes oxidized [39,42]. Additionally, the pro-inflammatory effects of HMGB1 are also dependent on its redox state [43–46]. We have previously shown that oxaliplatin treatment induces oxidative stress through the upregulation of inducible nitric oxide synthase (iNOS) within the LMMP, as well as an increase in mitochondrial superoxide production and protein nitrosylation in myenteric neurons [9]. Given that oxaliplatin induces an oxidative environment, this particular DAMP may therefore be subjected to oxidisation, and thus, have its inflammatory potential blunted; this needs further investigation. Moreover, we observed the downregulation of TLR7 and TLR9 following oxaliplatin treatment, with no changes to TLR2, TLR3 or TLR4. TLRs are membrane-bound receptors which recognise a myriad of ligands produced by microbiota, as well as DAMPs [47–49]. TLR stimulation by ligands triggers signal transduction pathways and immunological responses [50,51]. TLR2 recognises bacterial lipoproteins and non-enterobacterial LPS [52]. Furthermore, TLR3 recognises double-stranded viral RNA, and TLR4 is stimulated by classical LPS [53,54]. TLR7 recognises single-stranded viral RNA, whereas TLR9 is stimulated by bacterial and viral DNA [51,55,56]. The downregulation of TLR7 and TLR9 observed in this study would impair recognition of certain pathogens and prospective immune responses.

Moreover, the downregulation of H2-D1 was observed following oxaliplatin treatment. The H2-D1 gene is associated with MHC-related molecules and antigen presentation. This downregulation could impact antigen loading and presentation to effector lymphocytes such as CD8^+^ T cells. Oxaliplatin treatment has been shown to evoke changes in another DAMP, calreticulin, a multifunctional endoplasmic reticulum and nuclear envelope-resident protein [28,57]. A major function for calreticulin is the biogenesis and correct folding and assembly of MHC-related molecules [58,59]. The downregulation of H2-D1 may be associated with changes in calreticulin expression which requires further investigation. The lack of gastrointestinal immune responses following oxaliplatin treatment could be due to defective MHC assembly and antigen presentation.

The mammalian gastrointestinal tract is colonised by diverse microorganisms where a symbiotic relationship between the host and microbiota exists [60]. The composition of microbiota throughout the gastrointestinal tract can vary depending on the location. The colon in particular has the greatest microbial density with an astounding 1x 10^12^ organisms per gram of feces (dry weight) [61]. In this study, we isolated fecal DNA and identified five major phyla which included: *Bacteroidetes*, *Deferribacteres*, *Firmicutes*, *Proteobacteria* and *Tenericutes*. Oxaliplatin did not cause significant changes to the microbiota at the phylum level in terms of OTUs, Chao richness and diversity, however, induced a significant reduction in *Parabacteroides* and *Prevotella*_*1*_ species, but caused an increase in *Prevotella*_*2*_ and *Odoribacter*. *Parabacteroides*, *Prevotella* and *Odoribacter* genera stem from the *Bacteroides* phylum. The *Bacteroides* phyla and their successive genera stem from the *Bacteroidetes* family. These bacteria are gram-negative, anaerobic, non-spore-forming rods which are commensal to the gastrointestinal tract, but are known to be opportunistic pathogens in circumstances of intestinal barrier destruction [62]. It is well established that anti-cancer agents cause considerable damage to the gastrointestinal mucosa which can act as a gateway for microbiota-induced inflammation throughout the colon [10,63,64]. Gram-negative bacteria produce endotoxins such as lipopolysaccharides (LPS; bacterial cell wall constituents) which can induce immunological responses and contribute to inflammatory diseases through TLR binding [65–68]. LPS come in ‘classical’ and ‘non-classical’ forms, depending on its structural configuration and degree of acylation [69–71]. Classical LPS are acylated lipid A molecules on short-chain fatty acids which stimulate a number of pro-inflammatory responses through cytokine production (predominantly TNF-α and IL-6) by innate immune cells (neutrophils, monocytes and macrophages) [69,72]. TLR4 is a main receptor for ‘classical’ LPS produced by *Escherichia coli*, whereas LPS from *Bacteroides* species are considered ‘non-classical’ and thus, TLR binding may not induce a rapid or strong immunological response [69,73]. Although structurally similar to classical LPS, the non-classical version still contains a lipid A centre, however, with varying degrees of acylation, and is often attached to long-chain fatty acids linked to amino-sugar backbones which is thought to hinder LPS recognition and signalling [65,69]. The inability to produce potent or classical LPS by the *Bacteroides* family, and the lack of changes to total TLR4 immunoreactivity observed in our present study may explain the absence of inflammation within the colon despite the increased abundance of such species at the genus level following oxaliplatin treatment.

It is known that immune cells within the gastrointestinal mucosa function differently to their counterparts found within the circulation. The mucosal immune system has co-evolved with the gastrointestinal microbiota/antigens and sterile inflammation to downregulate pro-inflammatory responses whilst uncompromising microbicidal or phagocytic activity [74,75]. No inflammation (determined by CD45^+^ immune cell infiltrate) or differences in total TLR4 immunoreactivity throughout the thickness of the colon was observed following oxaliplatin treatment.

There were no noticeable effects on MPO activity within the colon following oxaliplatin treatment. MPO is a well-established biomarker of inflammation in various conditions such as multiple sclerosis, ischaemic heart disease and acute coronary syndromes, as well as ulcerative colitis [76–80]. MPO is a cytotoxic constituent released by activated myeloid cells such as neutrophils, which also has micobicidal capacity [81–84]. Despite changes in gastrointestinal microbiota at the genus level, as well as increased HMGB1 expression which is known to have pro-inflammatory effects, MPO activity was not altered. Thus, our MPO data provide further evidence that oxaliplatin does not induce gastrointestinal inflammation, which presumably, would not have major effects on the colon myenteric plexus.

Furthermore, we demonstrate that oxaliplatin treatment downregulated gene expression for the pro-inflammatory cytokines IL-1β, IL-12β within the colon. Cytokines can mediate inflammation and trigger extrinsic apoptotic cascades. Although the extrinsic and intrinsic apoptotic pathways are considered to be separate entities, there is some cross over between the two signal transduction pathways [85–87]. Given that pro-inflammatory cytokines are downregulated in the colon following oxaliplatin treatment it is unlikely that the extrinsic apoptotic cascade is implicated in the underlying mechanism of cell death within the myenteric plexus. It has previously been shown that oxaliplatin treatment does not alter IL-1β, IFN-γ expression within rat spinal cord and DRG neurons, nor does it induce immune cell infiltration [88]. However, there are some conflicting studies which have demonstrated a significant increase in pro-inflammatory cytokines IL-1β and TNF-α within the rat spinal cord following oxaliplatin treatment, and a reduction in the anti-inflammatory cytokines IL-4 and IL-10 [89–91]. In the aforementioned studies, acute experiments were conducted on rats which were treated with oxaliplatin through a single 6mg/kg/d, or a 10mg/kg/d for 5 consecutive days. This is in contrast to our current study where we have chronically treated mice tri-weekly for up to 14 days with a 3mg/kg/d. The differences between oxaliplatin dosage, species and experimental time points may contribute to varying results.

Furthermore, we showed that the chemokine Ccl2 was upregulated within the colon following oxaliplatin treatment. Despite the increase in Ccl2 (a monocyte chemoattractant), there were no increases in immune cells in the colon. Ccl2 is a pleiotropic ligand implicated in many pathways. Ccl2 has demonstrated a role in shaping pro-inflammatory/anti-inflammatory macrophage responses as a deficiency leads to skewed pro-inflammatory phenotypes producing high levels of IL-6 and TNF-α [92]. The upregulation of Ccl2 in the colon following oxaliplatin treatment may play a role in dampening pro-inflammatory cytokine production by promoting the polarisation of anti-inflammatory macrophages. Ccl2 is also upregulated in response to gastrointestinal microbiota [93]. Whether the increase in microbiota species observed in this study is implicated in Ccl2 upregulation requires further study. Furthermore, previous research has demonstrated that Ccl2 is upregulated in DRG neurons and microglia following peripheral nerve injury prompting macrophage infiltration and sensory neuropathy [94–96]. Ccl2 can also be upregulated by other cytokines such as s100β [97]. We have previously demonstrated that s100β expression within the myenteric plexus of the ileum is increased following oxaliplatin treatment (Robinson et al., 2016). Whether upregulated s100β can impact Ccl2 levels within the colon requires further investigation. In addition, this study demonstrates that oxaliplatin treatment downregulated Ccl5 and Ccl22. Ccl5 plays a role in lymphocyte trafficking and promoting T cell polarisation towards an IFN-γ-producing Th1 phenotype [98]. Moreover, Ccl22 is implicated in lymphocyte and eosinophil migration [99,100]. The downregulation of Ccl5 and Ccl22 following oxaliplatin treatment may impact lymphocyte and/or eosinophil migration throughout the colon.

Downregulation of Aicda and Csf2 were also observed following oxaliplatin treatment. Aicda regulates B cell proliferation and immunoglobulin class switching [101]. In this study we did not observe any changes to B cell populations within the PPs and MLNs following oxaliplatin treatment. It is unknown whether the downregulation of this gene may affect B cell numbers and function at a later time point. Csf2 is a cytokine involved in macrophage and granulocyte production and maturation [102,103]. Downregulation of Csf2 is consistent with the decrease in macrophages and dendritic cells within the MLNs observed in this study.

We investigated the effects of oxaliplatin treatment on the immunological responses within the PPs and MLNs. PPs contain specialised epithelia known as microfold or M cells which are pivotal induction sites for pathogen or antigen-specific immune responses [104,105]. Dendritic cells within the PPs consistently sample luminal antigens and bacteria, and if loaded with an inflammatory stimulus, prime local T cells to initiate a response [27,106,107]. Intestinal dendritic cells also migrate to T cell areas of MLNs which is thought to play a role in maintaining immunological tolerance [108]. Although there were no demonstrable changes to the proportion of immune populations within the PPs following oxaliplatin treatment, a proportional reduction in both macrophages and dendritic cells within the MLNs was observed. Immunological responses within the lymph nodes would typically occur once antigen presenting cells are antigen-loaded and have migrated to prime T cells to initiate an immune response. However, no demonstrable changes to T cell populations were observed within the PPs or MLNs following oxaliplatin treatment.

Furthermore, oxaliplatin treatment did not induce changes to other immune cell populations investigated in this study which include eosinophils, NK cells, γδ T cells, B cells, CD4^+^ T cells, CD8^+^ T cells and NKT cells, all of which have important roles in maintaining gastrointestinal homeostasis as well as initiating and modulating immune responses.

Under normal conditions eosinophils reside within haematopoietic and lymphatic tissues, as well as the gastrointestinal tract mucosa [109,110]. Eosinophils are pro-inflammatory effector cells which release pleiotropic chemokines, cytokines and cytotoxic granules such as eosinophil peroxidase and eosinophil-derived neurotoxin [111,112]. Eosinophils have been implicated in a number of inflammatory conditions in the gastrointestinal tract. These include eosinophilic gastroenteritis, allergic colitis, and inflammatory bowel disease [113–115]. In this study we did not observe changes in the proportion of eosinophils within the PPs and MLNs which is consistent with oxaliplatin-induced immunosuppression observed in the colons from oxaliplatin-treated mice. Thus, eosinophils do not appear to be affected by oxaliplatin treatment, and are unlikely to mediate enteric neuropathy.

Furthermore, no changes in the proportion of NK cells within the PPs and MLNs were observed following oxaliplatin treatment. NK cells are innate lymphocytes present throughout the gastrointestinal tract and associated lymphoid organs [116]. NK cells primarily defend against viral infections, tumors, and microbial species through cell-mediated cytolytic processes involving perforin and granzyme molecules [116–119]. Although oxaliplatin treatment caused mucosal injury, microbial dysbiosis, the proportion of NK cells remained unaffected. Additionally, the presentation of DAMPs following oxaliplatin treatment does not appear the affect the proportion of NK cells within the PPs and MLNs. A study investigating the effects of oxaliplatin treatment on ovarian cancer cells has shown that this platinum-based drug increases NK cell-mediated toxicity [120]. Analysis of NK cells in human peripheral blood of patients receiving low-dose cisplatin and 5-fluorouracil treatment has also shown to prevent NK cell suppression typically observed following colorectal surgery [121]. However, our data suggests that NK cell-mediated cytotoxicity is an unlikely cause for enteric neuropathy following oxaliplatin treatment. No immune cells infiltrated the myenteric plexus (determined by pan-leukocyte CD45^+^ labelling), and thus, cell-mediated killing of neurons and glia is not apparent.

γδ T cells are intraepithelial lymphocytes which act as immunosurveyers of the gastrointestinal tract [122]. These cells account for upto 50% of intraepithelial lymphocytes within the gastrointestinal tract and play a role in antigen presentation, anti-tumor immunity, microbial defense, neutrophil and macrophage recruitment and cytokine production [123–125]. We did not observe any changes in the proportion of γδ T cells in either the PPs or MLNs. Our data suggests that they are not infiltrating lymphoid organs to present antigens following oxaliplatin therapy, and that they are unlikely to be recruiting myeloid cells such as neutrophils and macrophages given that no observable changes in MPO activity within the colon was found. One study to date has demonstrated that oxaliplatin treatment induces infiltration of IL-17 producing γδ T cells to transplantable tumor sites [126]. It appears that oxaliplatin treatment can sensitise cancer cells to specific γδ T cell-mediated immunity, but this seems unlikely for enteric neurons, given that no infiltrating immune cells were observed.

B cells play an important role in intestinal immunity and mucosal tolerance. They are enriched within gastrointestinal associated lymphoid organs, synthesise IgA which functions to inhibit microbial adherence to mucosal surfaces, and neutralizes toxins, enzymes and antigens [127]. B cells also induce T cell dependent or independent responses. An *in vivo* study has shown that B cells can impede T cell responses characteristic of immunogenic cell death in mouse tumor model [128]. In our study we did not observe any changes in the proportion of B cells or various T cell subpopulations (CD4^+^, CD8^+^, NKT).

As there are a number of CD4^+^ T cell populations, such as Th1, Th2, Th9, Th17 and Tregs [129], we analyzed them collectively to get an overview of CD4^+^ T cell proportions following oxaliplatin treatment. CD4^+^ T cells influence innate and adaptive immune responses through conditioning the milieu with pro-inflammatory or anti-inflammatory cytokines and chemokines [130]. Although NKT cells are a subset of CD4^+^ T cells they are activated through CD1d-restricted lipid antigens as opposed to classical MHC class I and II molecules [131–133]. The most extensively studied lipid ligand for CD1d is α-galactosylceramide [133]. Both mammalian and bacterial lipids can stimulate NKT cells to stimulating rapid Th1 and Th2 responses. No changes were noted in the proportion of NKT cells within either the PPs or MLNs following oxaliplatin treatment. However, the effects of oxaliplatin treatment on NKT cells *in vivo* require further studies.

Furthermore, no demonstrable changes in the proportion of CD8^+^ T cells within the PPs or MLNs were shown following oxaliplatin treatment. CD8^+^ T cells are activated upon antigen presentation and can exert cytotoxic activity through two major pathways. These include the granule exocytosis or the death receptor (TNF-α and Fas) pathway [134,135]. Previous studies using peripheral blood and colon cancer cell lines have shown marked increases in T cell activation following the presentation of DAMPs induced by oxaliplatin treatment [28,136]. We have previously demonstrated that oxaliplatin treatment evokes the presentation of DAMPs within the myenteric plexus, however, no differences in T cell populations were observed.

Given that the gastrointestinal tract is constantly exposed to a myriad of antigens and pathogens, the local immune system has evolved over time to eradicate noxious stimuli through non-inflammatory host-defense mechanisms [74]. These data suggest that chemotherapy-induced microbiota dysbiosis, mucosal and neuronal damage in this case does not evoke inflammation or immunogenic cell death, thus, ENS damage is most likely mediated through direct drug toxicity.

## Conclusion

This is the first study to determine the effects of oxaliplatin treatment on the gastrointestinal microbiota and its effects on immune populations within the PPs and MLNs which mediate local inflammation. Oxaliplatin treatment does not induce severe inflammation throughout the thickness of the colon despite the presentation of DAMPs within in myenteric neurons. These data are suggestive of antigen and microbial-specific tolerance. Thus, it appears that oxaliplatin treatment is not associated with inflammatory enteric neuropathy, and further research is required to determine the mechanism of neuronal damage and death which contributes to gastrointestinal dysfunction.

## Supporting information

S1 Table Gene expression data generated using Mouse Cancer Inflammation and Immunity Crosstalk Array RT^2^ profiler PCR arrays. (DOC)

S1 Dataset Morphological changes in TLR4^+^ cells in the colon, and total number and immunoreactivity. TLR4^+^ cells in the colon were images at 100x magnification on a Nikon confocal microscope. We observed consistent differences in the morphology of TLR4^+^ cells in the oxaliplatin-treated group compared to the vehicle-treated cohort. 8 randomised images (20x magnification) per animal were used to count the number of TLR4^+^ cells in the colon, as well as immunoreactivity/image. Image J counter plugin was used to mark each cell to ensure they were only counted once, and we measured immunoreactivity/fluorescence by converting the image to 8-bit →binary→measure. We used % area result to determine immunoreactivity per image. The sum of TLR4^+^ cells and immunoreactivity was averaged between groups.
(XLSX)

S2 Dataset PCR Array of ‘Mouse Cancer Inflammation and Immunity Crosstalk’ (Qiagen, Cat. no. PAMM-181Z). All genes included in the array are listed, alongside quality control and normalisation expression. Changes in inflammation-associated gene expression are presented in heatmap and numerical formats. This dataset relates to all PCR data in this study, including Fig 4 (receptors) and Fig 7 (cytokines and chemokines).
(XLSX)

S3 Dataset 16s rRNA microbiota analysis following oxaliplatin treatment. Dataset includes numbers on microbiota phylum, class, order, family, and genus.
(XLS)

S4 Dataset Total number of CD45+ cells, immunoreactivity in the colon and MPO activity. Eight randomised images (20x magnification) per animal were used to count the number of CD45^+^ cells in the colon, as well as immunoreactivity/image. Image J counter plugin was used to mark each cell to ensure they were only counted once, and we measured immunoreactivity/fluorescence by converting the image to 8-bit →binary→measure. We used % area result to determine immunoreactivity per image. MPO activity was measured using the MPO Colorimetric Activity Assay (Sigma-Aldrich, Australia) according to manufacturers instructions.
(XLSX)

S5 Dataset Flow cytometry of PPs and MLNs. This data set contains raw values for all flow cytometry experiments on the various immune cell populations investigated in the PPs and MLNs.
(XLS)
