# Supplementary material for: Oxaliplatin-induced changes in microbiota, TLR4+ cells and enhanced HMGB1 expression in the murine colon
Source: PLoS One. 2018 Jun 12;13(6):e0198359. doi: 10.1371/journal.pone.0198359 (PMC5997344; doi:10.1371/journal.pone.0198359)
Supplement: S1 Table — (DOC) [file pone.0198359.s001.doc]

**Supplementary Material**

**Determining immunogenic cell death as a potential mechanism of oxaliplatin-induced enteric neuropathy**

**AUTHORS:** Vanesa Stojanovska^1^, Rachel M McQuade^1^, Sarah Fraser^1^, Monica Prakash^1^, Shakuntla Gondalia^2^, Rhian Stavely^1^, Enzo Palombo^3^, Vasso Apostolopoulos^4^, Samy Sakkal^1^* and Kulmira Nurgali^1^*

Table S1: Gene expression data generated using Mouse Cancer Inflammation and Immunity Crosstalk Array RT^2^ Profiler PCR arrays

| **Gene Symbol** | **Description** | **Av. C_T_ vehicle**  **treated** | **SD** | **Av. C_T_ oxaliplatin**  **treated** | | **SD** | **Fold Regulation** |
| --- | --- | --- | --- | --- | --- | --- | --- |
| **Ackr3** | Chemokine (C-X-C motif) receptor 7 | 26.67 | 0.03 | 26.69 | 0.20 | | -1.03 |
| **Aicda** | Activation-induced cytidine deaminase | 33.63 | 0.54 | 34.83 | 0.24 | | -2.32 |
| **Bcl2** | B-cell leukemia/lymphoma 2 | 27.67 | 0.09 | 27.47 | 0.08 | | 1.13 |
| **Bcl2l1** | Bcl2-like 1 | 27.07 | 0.00 | 26.84 | 0.17 | | 1.16 |
| **Ccl2** | Chemokine (C-C motif) ligand 2 | 29.59 | 0.14 | 27.87 | 0.24 | | 3.25 |
| **Ccl20** | Chemokine (C-C motif) ligand 20 | 26.67 | 0.10 | 27.13 | 0.08 | | -1.39 |
| **Ccl22** | Chemokine (C-C motif) ligand 22 | 31.54 | 0.25 | 32.92 | 0.45 | | -2.63 |
| **Ccl28** | Chemokine (C-C motif) ligand 28 | 27.93 | 0.05 | 27.59 | 0.08 | | 1.25 |
| **Ccl4** | Chemokine (C-C motif) ligand 4 | 34.2 | 0.15 | 33.96 | 0.32 | | 1.16 |
| **Ccl5** | Chemokine (C-C motif) ligand 5 | 29.56 | 0.06 | 30.67 | 0.09 | | -2.19 |
| **Ccr1** | Chemokine (C-C motif) receptor 1 | 28.77 | 0.15 | 29.15 | 0.16 | | -1.31 |
| **Ccr10** | Chemokine (C-C motif) receptor 10 | 30.4 | 0.07 | 29.95 | 0.02 | | 1.35 |
| **Ccr2** | Chemokine (C-C motif) receptor 2 | 28.88 | 0.02 | 28.79 | 0.27 | | 1.05 |
| **Ccr4** | Chemokine (C-C motif) receptor 4 | 32.8 | 0.28 | 33.04 | 0.55 | | -1.19 |
| **Ccr5** | Chemokine (C-C motif) receptor 5 | 30.26 | 0.05 | 30.45 | 0.02 | | -1.16 |
| **Ccr7** | Chemokine (C-C motif) receptor 7 | 30.18 | 0.04 | 30.3 | 0.25 | | -1.10 |
| **Ccr9** | Chemokine (C-C motif) receptor 9 | 30.6 | 0.22 | 31.21 | 0.02 | | -1.55 |
| **Cd274** | CD274 antigen | 29.46 | 0.00 | 29.77 | 0.03 | | -1.26 |
| **Csf1** | Colony stimulating factor 1 (macrophage) | 28.85 | 0.17 | 28.35 | 0.06 | | 1.40 |
| **Csf2** | Colony stimulating factor 2 (granulocyte-macrophage) | 33.48 | 0.13 | 34.36 | 0.39 | | -1.87 |
| **Csf3** | Colony stimulating factor 3 (granulocyte) | 35 | 0.00 | 35 | 0.00 | | -1.01 |
| **Ctla4** | Cytotoxic T-lymphocyte-associated protein 4 | 33.99 | 0.48 | 33.48 | 0.04 | | 1.41 |
| **Cxcl1** | Chemokine (C-X-C motif) ligand 1 | 33.05 | 0.04 | 33.09 | 0.05 | | -1.04 |
| **Cxcl10** | Chemokine (C-X-C motif) ligand 10 | 30.2 | 0.06 | 29.87 | 0.13 | | 1.24 |
| **Cxcl11** | Chemokine (C-X-C motif) ligand 11 | 33.45 | 0.74 | 34.1 | 1.24 | | -1.59 |
| **Cxcl12** | Chemokine (C-X-C motif) ligand 12 | 26.22 | 0.07 | 25.88 | 0.11 | | 1.25 |
| **Cxcl2** | Chemokine (C-X-C motif) ligand 2 | 34.57 | 0.61 | 34.57 | 0.61 | | -1.02 |
| **Cxcl5** | Chemokine (C-X-C motif) ligand 5 | 32.32 | 0.56 | 31.84 | 0.06 | | 1.38 |
| **Cxcl9** | Chemokine (C-X-C motif) ligand 9 | 34.63 | 0.19 | 34.42 | 0.71 | | 1.15 |
| **Cxcr1** | Chemokine (C-X-C motif) receptor 1 | 35 | 0.00 | 35 | 0.00 | | -1.01 |
| **Cxcr2** | Chemokine (C-X-C motif) receptor 2 | 34.8 | 0.28 | 35 | 0.00 | | -1.16 |
| **Cxcr3** | Chemokine (C-X-C motif) receptor 3 | 32.02 | 0.03 | 32.34 | 0.09 | | -1.26 |
| **Cxcr4** | Chemokine (C-X-C motif) receptor 4 | 27.48 | 0.14 | 27.36 | 0.00 | | 1.08 |
| **Cxcr5** | Chemokine (C-X-C motif) receptor 5 | 30.67 | 0.02 | 30.85 | 0.22 | | -1.15 |
| **Egf** | Epidermal growth factor | 31.13 | 0.10 | 31.96 | 0.07 | | -1.80 |
| **Egfr** | Epidermal growth factor receptor | 28.05 | 0.00 | 27.87 | 0.11 | | 1.12 |
| **Fasl** | Fas ligand (TNF superfamily, member 6) | 33.09 | 0.42 | 33.43 | 0.27 | | -1.28 |
| **Foxp3** | Forkhead box P3 | 33.99 | 0.14 | 33.32 | 0.68 | | 1.57 |
| **Gbp2b** | Guanylate binding protein 1 | 27.54 | 0.08 | 27.37 | 0.07 | | 1.12 |
| **Gzma** | Granzyme A | 31.08 | 0.24 | 31.72 | 0.05 | | -1.58 |
| **Gzmb** | Granzyme B | 32.81 | 0.63 | 32.72 | 0.12 | | 1.05 |
| **H2-D1** | Histocompatibility 2, D region locus 1 | 33.86 | 0.08 | 35 | 0.00 | | -2.23 |
| **H2-K1** | Histocompatibility 2, K1, K region | 26.18 | 0.12 | 26.21 | 0.23 | | -1.03 |
| **Hif1a** | Hypoxia inducible factor 1, alpha subunit | 25.13 | 0.04 | 24.95 | 0.15 | | 1.13 |
| **Ido1** | Indoleamine 2,3-dioxygenase 1 | 31.74 | 0.03 | 32.1 | 0.04 | | -1.30 |
| **Ifng** | Interferon gamma | 34.19 | 0.59 | 34.95 | 0.07 | | -1.71 |
| **Igf1** | Insulin-like growth factor 1 | 27.11 | 0.10 | 27.48 | 0.07 | | -1.31 |
| **Il10** | Interleukin 10 | 34.63 | 0.02 | 35 | 0.00 | | -1.31 |
| **Il12a** | Interleukin 12A | 31.53 | 0.11 | 31.49 | 0.10 | | 1.01 |
| **Il12b** | Interleukin 12B | 33.19 | 0.33 | 35 | 0.00 | | -3.56 |
| **Il13** | Interleukin 13 | 34.45 | 0.30 | 34.65 | 0.16 | | -1.17 |
| **Il15** | Interleukin 15 | 27.59 | 0.06 | 27.96 | 0.12 | | -1.31 |
| **Il17a** | Interleukin 17A | 35 | 0.00 | 35 | 0.00 | | -1.01 |
| **Il1a** | Interleukin 1 alpha | 31.97 | 0.30 | 32.18 | 0.39 | | -1.17 |
| **Il1b** | Interleukin 1 beta | 29.02 | 0.07 | 30.02 | 0.15 | | -2.02 |
| **Il1r1** | Interleukin 1 receptor, type I | 27.89 | 0.10 | 27.54 | 0.08 | | 1.26 |
| **Il2** | Interleukin 2 | 35 | 0.00 | 35 | 0.00 | | -1.01 |
| **Il22** | Interleukin 22 | 35 | 0.00 | 35 | 0.00 | | -1.01 |
| **Il23a** | Interleukin 23, alpha subunit p19 | 34.8 | 0.28 | 34.06 | 0.06 | | 1.65 |
| **Il4** | Interleukin 4 | 33.04 | 0.17 | 33.78 | 0.64 | | -1.69 |
| **Il5** | Interleukin 5 | 34.79 | 0.30 | 34.74 | 0.37 | | 1.02 |
| **Il6** | Interleukin 6 | 34.83 | 0.24 | 34.77 | 0.32 | | 1.03 |
| **Irf1** | Interferon regulatory factor 1 | 25.68 | 0.02 | 25.58 | 0.11 | | 1.06 |
| **Kitl** | Kit ligand | 24.63 | 0.07 | 24.41 | 0.07 | | 1.14 |
| **Mif** | Macrophage migration inhibitory factor | 23.77 | 0.10 | 23.66 | 0.10 | | 1.07 |
| **Myc** | Myelocytomatosis oncogene | 26.66 | 0.07 | 26.57 | 0.08 | | 1.05 |
| **Myd88** | Myeloid differentiation primary response gene 88 | 30.62 | 0.14 | 30.66 | 0.15 | | -1.04 |
| **Nfkb1** | Nuclear factor of kappa light polypeptide gene enhancer in B-cells 1, p105 | 26.52 | 0.06 | 26.43 | 0.13 | | 1.05 |
| **Nos2** | Nitric oxide synthase 2, inducible | 31.1 | 0.02 | 31.3 | 0.10 | | -1.16 |
| **Pdcd1** | Programmed cell death 1 | 34.5 | 0.71 | 34.59 | 0.39 | | -1.07 |
| **Ptgs2** | Prostaglandin-endoperoxide synthase 2 | 28.58 | 0.06 | 29.22 | 0.04 | | -1.57 |
| **Spp1** | Secreted phosphoprotein 1 | 30.13 | 0.08 | 30.15 | 0.00 | | -1.03 |
| **Stat1** | Signal transducer and activator of transcription 1 | 26.56 | 0.00 | 26.47 | 0.13 | | 1.05 |
| **Stat3** | Signal transducer and activator of transcription 3 | 25.6 | 0.00 | 25.58 | 0.15 | | 1.00 |
| **Tgfb1** | Transforming growth factor, beta 1 | 27.1 | 0.03 | 27.11 | 0.08 | | -1.02 |
| **Tlr2** | Toll-like receptor 2 | 28.62 | 0.03 | 28.59 | 0.21 | | 1.01 |
| **Tlr3** | Toll-like receptor 3 | 26.89 | 0.05 | 26.94 | 0.12 | | -1.05 |
| **Tlr4** | Toll-like receptor 4 | 27.42 | 0.07 | 27.39 | 0.10 | | 1.01 |
| **Tlr7** | Toll-like receptor 7 | 29.72 | 0.35 | 30.55 | 0.12 | | -1.81 |
| **Tlr9** | Toll-like receptor 9 | 29.94 | 0.18 | 30.92 | 0.14 | | -2.01 |
| **Tnf** | Tumor necrosis factor | 31.1 | 0.13 | 31.12 | 0.48 | | -1.03 |
| **Tnfsf10** | Tumor necrosis factor (ligand) superfamily, member 10 | 28.69 | 0.02 | 29.02 | 0.07 | | -1.27 |
| **Trp53** | Transformation related protein 53 | 27.72 | 0.12 | 27.54 | 0.08 | | 1.12 |
| **Vegfa** | Vascular endothelial growth factor A | 27.04 | 0.02 | 26.5 | 0.16 | | 1.44 |

Gene symbol and description of the 84 inflammation and immunity-related genes present on the PCR array. Differential gene expression is given as fold up- or down-regulation in the oxaliplatin-treated group relative to the vehicle-treated group; data represent the mean of duplicate determinations. Mean C_T_ values ± SD. A C_T_ value of 35 is considered to be the cut-off for reliable detection of gene expression

.
